# Controlled intracellular aggregation of magnetic particles improves permeation and retention for magnetic hyperthermia promotion and immune activation

**DOI:** 10.7150/thno.80821

**Published:** 2023-02-27

**Authors:** Ao Hu, Yiyao Pu, Na Xu, Zhongyuan Cai, Ran Sun, Shengxiang Fu, Rongrong Jin, Yingkun Guo, Hua Ai, Yu Nie, Xintao Shuai

**Affiliations:** 1 National Engineering Research Center for Biomaterials, Sichuan University, Chengdu, 610064, P. R. China; 2 College of Biomedical Engineering, Sichuan University, Chengdu, 610065, P. R. China; 3 Development and Related Diseases of Women and Children Key Laboratory of Sichuan Province, West China Second University Hospital, Sichuan University, Chengdu 610041, P. R. China; 4 Nanomedicine Research Center, The Third Affiliated Hospital of Sun Yat-sen University, Guangzhou 510630, China

**Keywords:** Intracellular aggregation, magnetic hyperthermia therapy, immune activation, giant cell, magnetic resonance imaging

## Abstract

**Rationale**: Magnetic nanoparticles (MNPs) are the most used inorganic nanoparticles in clinics with therapeutic and imaging functions, but the inefficient magneto-thermal conversion efficiency, fast leakage, and uneven distribution impair their imaging sensitivity and therapeutic efficacy in tumors.

**Methods**: Herein, we rationally designed a system containing pH-controllable charge-reversible MNPs (M20@DPA/HA) and negatively charged MMPs with different sizes (M5 and M20), which could induce intracellular aggregation. The dynamic hydrazone bonds with pH controllability were formed by the surface hydrazides on MNPs and aldehydes of hyaluronic acid (HA). Under the acidic pH, intracellular aggregation of the complex composed by M20@DPA/HA and M5 (M5&20), or M20@DPA/HA and M20 (M20&20) were investigated. In addition, the magnetic hyperthermia therapy (MHT) efficiency of tumor cells, tumor-associated macrophages polarization, giant cells formation and immune activation of tumor microenvironment were explored via a series of cell and animal model experiments.

**Results**: Through physical and chemical characterization, the aggregation system (M20&20) exhibited a remarkable 20-fold increase in magnetothermal conversion efficiency compared to individual MNPs, together with enhanced penetration and retention inside the tumor tissues. In addition, it could promote immune activation, including repolarization of tumor-associated macrophages, as well as the formation of giant cells for T cell recruitment. As a result, the M20&20 aggregation system achieved a high degree of inhibition in 4T1 mouse mammary tumor model, with little tumor growth and metastasis after magnetic hyperthermia therapy.

**Conclusions**: A controlled intracellular aggregation system was herein developed, which displayed an aggregation behavior under the acidic tumor microenvironment. The system significantly enhanced MHT effect on tumor cells as well as induced M1 polarization and multinucleated giant cells (MGC) formation of TAM for immune activation. This controlled aggregation system achieved barely tumor growth and metastasis, showing a promising strategy to improve MNPs based MHT on deteriorate cancers.

## Introduction

Magnetic nanoparticles (MNPs) have a wide array of biomedical applications, including magnetic resonance imaging (MRI), magnetofection, magnetic hyperthermia therapy (MHT), drug delivery, ferroptosis of cancer cells, polarization of macrophages and immunotherapy.^1^-^8^ Although MNPs have been successfully used in the clinic as MRI contrast agents and mediators of MHT, their application still exhibits challenges in unsatisfactory imaging sensitivity and therapeutic efficacy, due to diffuse leakage and uneven distribution. 9

According to reports, size elevation of MNPs can improve *T*_2_ relaxivity and magneto-thermal conversion efficiency (with a high specific absorption rate, SAR). 10, 11 However, an unrestricted increase of the MNPs size could induce fast clearance by the reticuloendothelial system, and reduce particle penetration into the tumor, which led to the confinement of MNPs in the surface layer of tissue. 12 Therefore, the controllable aggregation is necessary for unimpeded circulation in the systemic cycle, improved penetration, and enhanced accumulation at target tissues. Until now, some inducible aggregation strategies have already shown a positive effect in MRI. 11, 13 The reduction-activated, caspase 3/7-instructed, and transglutaminase-controlled aggregation of MNPs substantially increased the retention of MRI contrast agents with enhanced *T*_2_-weighted imaging 14-16. However, few studies have focused on the MHT enhancement and related therapeutic efficiency of aggregated MNPs, such as ferroptosis, polarization, and other immune activation. And low pH as a more common and typical tumor microenvironment has not yet been applied as the trigger for MNPs aggregation.

It is worth noting that intracellular accumulation of MNPs has been demonstrated a better MHT effect. 17 Some researchers noted that intracellular accumulation of MNPs has a better MHT effect, which might because more MNPs particles can be retained after cell endocytosis, reducing the loss of particles between cells 18, 19. Meanwhile, several theoretical and experimental studies showed that more intracellular accumulation could not only better regulate the polarization of tumor-associated macrophages (TAM) for altering the tumor microenvironment, 20, 21 but also induce stronger Fenton reactions to generate reactive oxygen species (ROS) for tumor cell killing 22.

**Figure 1:**
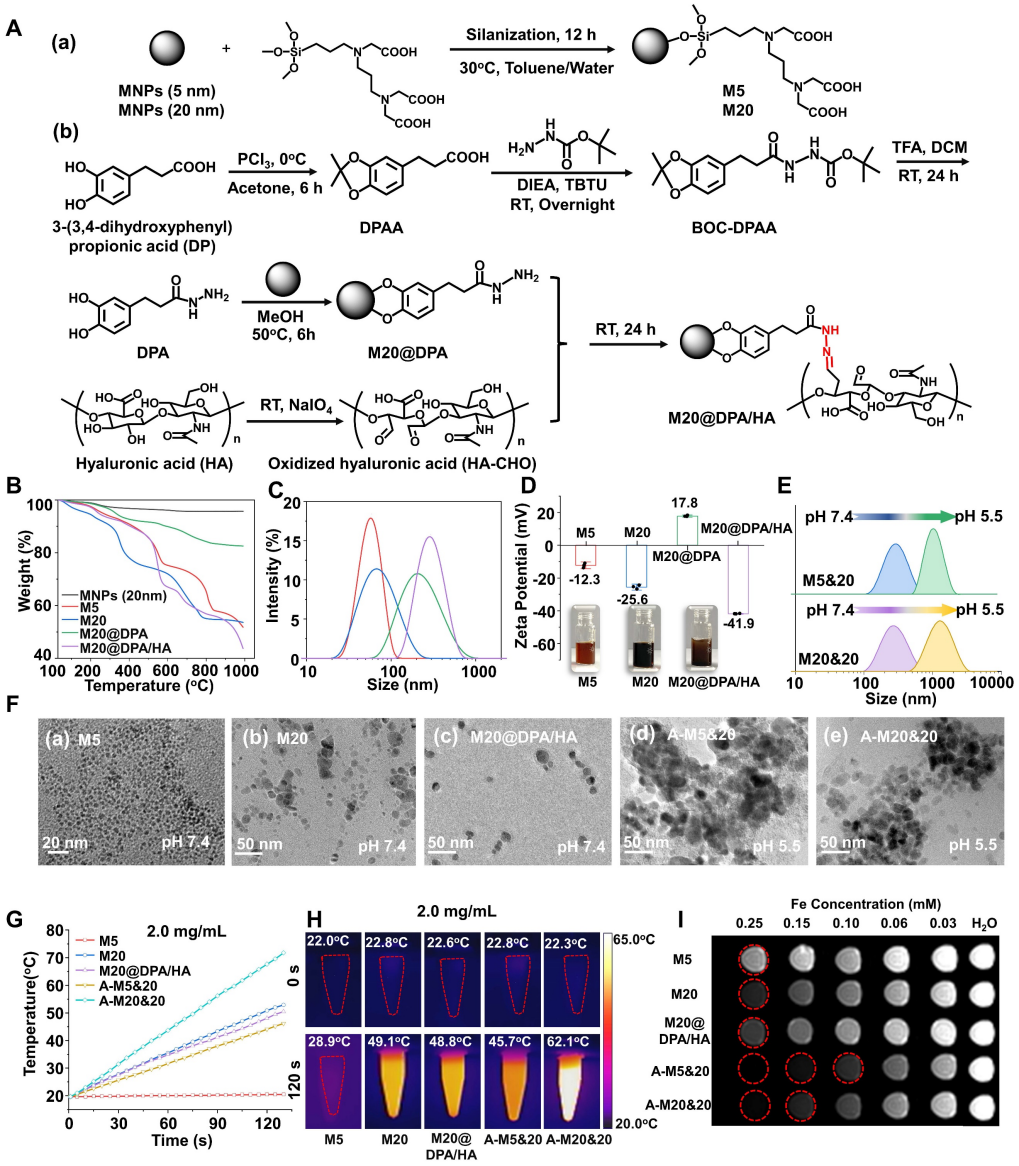
Chemical design and characterization of the MNPs with pH-sensitive charge reversion for controlled intracellular aggregation. (A) Synthesis process of various MNPs in the aggregation system. (a) Negatively charged MNPs with particle size of 5 nm (M5) and 20 nm (M20), (b) synthesis of charge reversible MNPs (M20@DPA/HA) with oxidized hyaluronic acid (HA-CHO) coating and hydrazone linkage. (B) Thermogravimetric (TGA) curve of various MNPs. (C) Z-average size, zeta potential and optical photo (D) of M5, M20, M20@DPA and M20@DPA/HA, respectively. (E) Size change of M5&20 and M20&20 with different pH. (F) Morphology of various MNPs by TEM observation at pH 7.4 (a-c) and 5.5 (d and e). When HA was drop off in the extracellular acidic condition, positively charged M20@DPA would aggregate with negatively charged M5 or M20 via electrostatic interactions to form A-M5&20 or A-M20&20. (G) Temperature change curves of individual MNPs and their aggregations under alternating magnetic field (AMF) (2 mg Fe/mL, 15 KA/m, 300 kHz). (H) Infrared thermal imaging of MNPs (2 mg/mL) under AMF for 120 s. (I) *T*_2_-weighted images of different MNPs and aggregations dispersed in 1% agarose gel at indicated concentrations under a 7.0 T magnetic field. The scale bars are 20 and 50 nm, respectively.

Thus, it is hypothesized that the controlled intracellular aggregation of MNPs may induce a better therapeutic effect, resulting from magnetic hyperthermia, or/and immune activation. As a proof of concept, we designed an MNPs system (**Figure 1A**) consisting of negatively charged MNPs (M5 & M20) with different sizes and pH-responsive charge reversal MNPs (M20@DPA/HA). We hypothesize that, under acidic conditions (in the endosome/lysosome), the pH-responsive linker hydrazone bond in M20@DPA/HA is expected be fractured, leading to the decoating of negatively charged hyaluronic acid and re-exposure of cationic MNPs. Thus, the negatively charged M5 or M20 can interact with the positively charged M20@DPA for aggregation. The controlled aggregation process, location, and related effect on cellular accumulation were investigated. The permeation and retention, specific immune activation, and MHT effect were further evaluated in an *in situ* breast tumor model.

## Results and Discussion

### Preparation and characterization of varied MNPs

In order to explore the effect of aggregates on MHT and MRI performance, MNPs of 5 and 20 nm were first prepared with different methods (**Figure S1A**). 23, 24 N-[(3-trimethoxysilyl) propyl] ethylenediamine triacetic acid trisodium salt with carboxyl groups was then coupled with MNPs for the surface modification. After silanization (**Figure 1A (a)**), a peak of 545 cm^-1^ (Fe-O) 25, 26, absorption bands around 1100 and 1000 cm^-1^ (Si-O-Si) 24, 27 and carboxylate absorption bands 24, 26 at 1628 and 1401 cm^-1^ were displayed in the Fourier transform infrared (FTIR) spectrum (**Figure S1B**), which demonstrated the formation of negatively charged MNPs (M5 & M20). As shown in **Figure S1C**, M5, M20 and M20@DPA/HA exhibited superparamagnetic behavior without magnetic hysteresis and remanence, which demonstrated that the Fe_3_O_4_ core was intact during the surface modification. The saturation magnetizations of M5, M20 and M20@DPA/HA were 45, 85 and 78 emu/g, respectively. In the synthesis of charge-reversible MNPs, DPA with a structure of catechol was first conjugated to MNPs (20 nm) (**Figure 1A (b)**), due to its strong affinity to metal oxide nanocrystals. When the vicinal hydroxyl groups of hyaluronic acid (HA) were oxidized to be HA-CHO 28-30 (**Figure 1A (b)**), the hydrazide groups on the other end of DPA could react with the aldehyde groups from HA-CHO to form a dynamic bond. The characteristic absorbance peak at 1636 cm^-1^ in FTIR spectrum (**Figure S1B**) was attributed to the imine bond (C=N) of M20@DPA/HA. 31 The thermal gravimetric analyzer (TGA) weight losses of various MNPs (M5, M20, M20@DPA, and M20@DPA/HA) were 48.2%, 46.4%, 17.45%, and 56.1%, respectively (**Figure 1B**). Through the TGA detection, the weight ratio of M20, DPA and HA in M20@DPA/HA were 100: 13: 52 (M20: DPA: HA). And the average hydrodynamic particle size of M5, M20, M20@DPA, and M20@DPA/HA were 58.8, 68.1, 190.0, and 295.3 nm (**Figure 1C**), with prospected zeta potential (-12.3, -25.6, 17.8, and -41.9 mV) (**Figure 1D**).

### pH-responsive aggregation formation of varied MNPs

The mixture of M20@DPA/HA and M5 (or M20) was incubated at different pH conditions to evaluate the controlled aggregation. At neutral pH 7.4, the average sizes of M5&20 and M20&20 both remained the same at ~ 298 nm within 5 h (**Figure S4A and B**). In contrast, the hydrodynamic diameter of M5&20 and M20&20 increased 2 folds in pH 6.8 and reached a micrometer level in pH 5.5 after 5 h incubation (**Figure 1E**). Transmission electron microscopy (TEM) images also intuitively displayed the original well-distributed M5, M20, and M20@DPA/HA as well as the induced aggregation of M5&20 and M20&20 (**Figure 1F**, **S4C and D**). In addition, samples of mixed MNPs in pH 5.5 were used to simulate the intracellular aggregates, named A-M5&20 or A-M20&20. This pH-responsive aggregation guaranteed stability of the MNPs system in the extracellular matrix and fast aggregation in acidic intracellular organelles (such as lysosomes) with a low pH microenvironment.

### Magneto-thermal conversion efficiency and MRI performance of varied MNPs

We conducted experiments to verify whether our predicted aggregation benefits the promotion of MHT and MRI, using the SAR as an indicator of thermal conversion efficiency, and *T*_2_ relaxivity (*r*_2_) values to represent imaging performance. 32, 33 According to heating curves (**Figure 1G, S5A and B**), the calculated SAR values of M5, M20, M20@DPA/HA, A-M5&20, and A-M20&20 were 41.8, 560.1, 547.6, 413.8, and 844.4 W/g, respectively (**Table S2**). The SAR showed a size-dependent increase, that values of M20 and M20@DPA/HA with larger sizes were about 100 folds higher than that of M5. 9, 34 After aggregation, A-M20&20 displayed the highest SAR. The phenomena were consistent with previous studies, in which the increased particle sizes and appropriate aggregation of MNPs could enhance the magneto-thermal conversion efficiency 11, 32 and *T*_2_ relaxivity 35. Interestingly, A-M5&20 exhibited a lower SAR value compared with M20@DPA/HA, which is probably due to the very low magneto-thermal conversion efficiency of M5. A similar result was obtained from infrared thermal imaging photos after magnetic heating (**Figure 1H, S5D and E**). As shown in **Figure S6**, the temperature of PBS and DMEM did not change significantly after magnetic heating for 20 min, which indicated that the temperature of solvents will not rise when exposure to AMF. MRI scan of MNPs was performed to acquire *T*_2_-weighted images and *r*_2_ values. As shown in **Figure 1I and S5C**, A-M5&20 (579.6 mM^-1^s^-1^) and A-M20&20 (465.1 mM^-1^s^-1^) displayed higher *r*_2_ values than the single MNPs with nearly 2 folds increase. Interestingly, the *r*_2_ value of A-M5&20 with different-sized monomers was higher than that of A-M20&20 with the same-sized monomer. The possible reason is that aggregation of different particle sizes had a greater impact on field inhomogeneity than the aggregation of the same particle size. 35

### pH-responsive intracellular aggregation of MNPs for the enhancement of MHT

**Figure 2:**
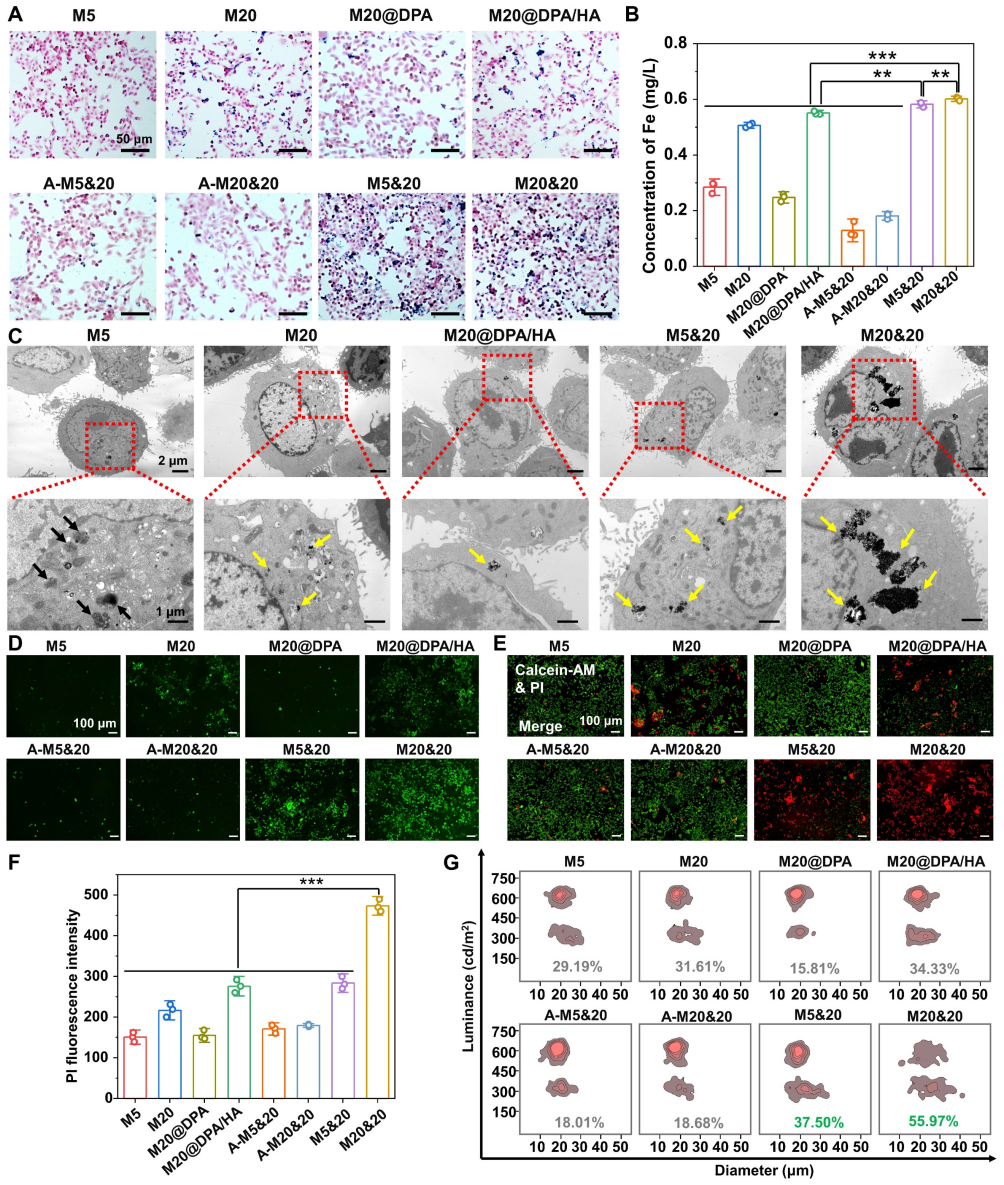
Controlled intracellular aggregation and killing effect of MNPs in 4T1 cells. Cellular uptake (A) and intracellular Fe concentrations (B) of MNPs by Prussian blue staining and ICP-OES analysis after 24 h incubation. The scale bars are 50 μm. (C) TEM observation of intracellular accumulation after 24 h treatment with various MNPs. Black arrows: small size MNPs in endosome/lysosome, yellow arrows: intracellular aggregation of MNPs. The scale bars are 1 and 2 μm, respectively. (D) Generation of ROS after 24 h co-incubation with different MNPs under AMF (20 min) by fluorescent probe staining. Scale bars: 100 μm. (E) Live/dead staining with calcein acetoxymethyl ester (calcein-AM) and propidium iodide (PI) of cells after co-incubated with different MNPs under AMF. Scale bars: 100 μm (F) Fluorescence intensity of PI-stained 4T1 cells after being treated by various MNPs under AMF. (G) Dead cell ratio of different treatments by cell counting apparatus with trypan blue staining. ** P < 0.01, *** P < 0.001.

Some researchers believed that intracellular accumulation has higher MHT effects than extracellular deposition 18, 19, 36, 37, which might because more MNPs particles can be retained after cell endocytosis, reducing the loss of particles between cells. Thus, we conducted cellular uptake experiments to detect if the MNPs could aggregate inside the tumor cells, with Prussian blue staining and inductively coupled plasma optical emission spectrometry (ICP-OES) detection of breast cancer (4T1) cells (**Figure 2A and B**). The M5&20, M20&20 mixture treated cells showed the highest intracellular iron content. It probably resulted from the formation of MNPs aggregates inside cells, which reduced the exocytosis. On the contrary, the aggregates formed outside the cell (A-M5&20 and A-M20&20) were rarely internalized, since the large particles (> 1 μm) were hard for endocytosis. 38 The occurrence of intracellular aggregation was also observed by the TEM performance (**Figure 2C**), showing a large amount of MNPs aggregated inside the cytoplasm in M20&20-treated cells, followed by M20, M20@DPA/HA, and M5&20, while fewer MNPs were captured in endosome/lysosome in M5-treated cells.

With the highest cellular uptake and SAR, M20&20 induced the highest fluorescent signal of ROS in 4T1 cells compared with the other groups under AMF (**Figure 2D**). Because iron ions can induce the Fenton reaction and produce ROS to kill tumor cells 20, 39, which can be promoted by MHT. Without AMF, cells treated with the mixed MNPs produced more ROS signals than that treated with the monomer (**Figure S7A and B**), even with similar intracellular iron content. The possible reason is that the aggregates formed intracellularly are difficult to digest and exocytosis, which may lead to dysfunction of organelles and more stress responses. In addition, the live/dead staining (**Figure 2E and S7C**), quantitative flow cytometry detection (**Figure 2F**), and trypan blue assay (**Figure 2G**) illustrated that the mixture of M20&20 displayed the best treatment effect compared with other groups. Without AMF, the dead cell was hardly observed (**Figure S7D**), which indicated that all MNPs were relatively nontoxic. Besides, the aggregations of A-M5&20 and A-M20&20 induced seldom cell death, which might ascribe to the large size of nanoparticles reduced endocytosis of 4T1 cells. 38

### Giant cells formation and polarization of TAM induced by intracellular aggregation

**Figure 3:**
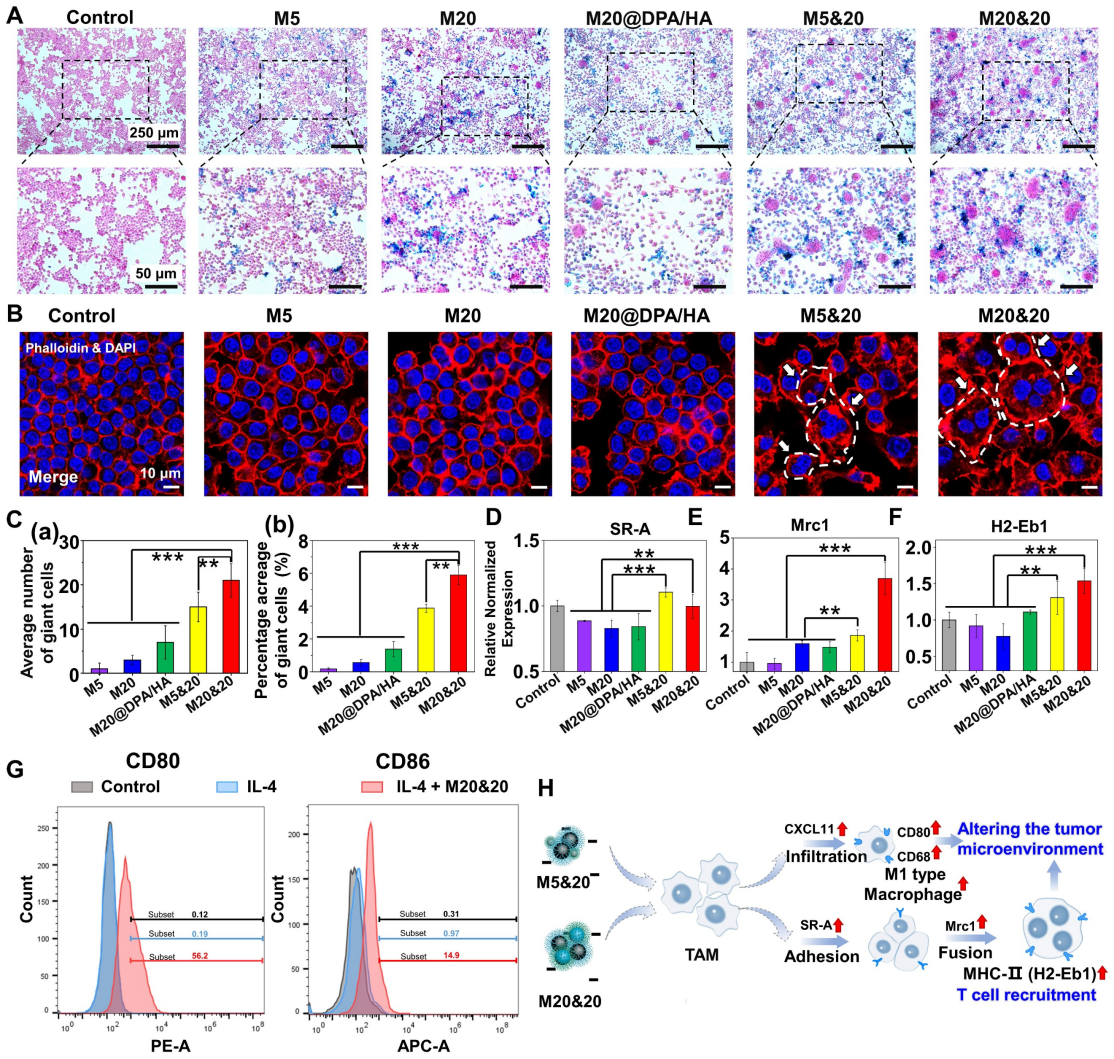
Immune activation in giant cells formation and macrophage polarization after treatments with different MNPs. (A) Cellular uptake of different MNPs after 24 h incubation on RAW264.7 by Prussian blue staining. Scale bars: 250 and 50 μm, respectively. (B) Detection of giant cell formation in RAW264.7 by cytoskeleton fluorescence staining with phalloidin (red) and DAPI (blue) after different treatments. Scale bars: 10 μm. (C) Average number (a) and percentage acreage (b) of the giant cells, calculated from the Prussian blue and cytoskeleton fluorescence staining in Figure 3A and B. Related gene evaluation for giant cell formation (D & E, SR-A, and Mrc1) and T cells recruitment (F, H2-Eb1) by quantitative real-time polymerase chain reaction (Q-PCR) analysis on RAW264.7 cells after incubation with different MNPs. (G) Flow cytometry analysis of CD80 and CD86 expression on IL-4 pretreated RAW264.7 cells incubated with M20&20. (H) Schematic of giant cell formation, macrophage polarization, and T cell recruitment, after treatment of M5&20 and M20&20, respectively. ** P < 0.01, *** P < 0.001.

In the following detection, the effect of aggregates on TAM was explored. A similar trend of macrophage uptake was observed among varied groups compared to tumor cells (**Figure 3A, S8A and B**). Interestingly, many cell clusters were observed in the macrophage cells after M5&20 and M20&20 treatment. Further verified by the fluorescent staining of phalloidin (red) and 4′,6-diamidino-2-phenylindole (DAPI) (blue), macrophages clusters had fused into the multinucleated giant cells (MGC) (**Figure 3B and S8C**), which is responsible for digestion of larger particles that is difficult for single cells 40. By statistical analysis, the average number and percentage acreage of MGC were ~10-20 folds in the aggregated formed groups than in unformed ones in monomer-treated groups (**Figure 3C (a) and (b)**). Macrophages treated with M20&20 showed more and larger giant cell formation than M5&20, probably due to more intracellular aggregates accumulation. Much fewer MGCs were observed in the aggregates treated groups (A-M5&20 and A-M20&20, **Figure S8D and E**), resulting from their inefficient cell internalization. Besides, the upregulated expression of class A scavenger receptor (SR-A) and mannose receptor C-type 1 (Mrc1) further confirmed the giant cell formation, which was an indicator of macrophage adhesion and fusion, respectively (**Figure 3D and E**). More importantly, the increased expression of histocompatibility 2, class II antigen E beta (H2-Eb1) on giant cells was observed, which is expected to enhance the recognition and residence of CD4^+^ T helper cells against tumor cells (**Figure 3F**). 41, 42

We followingly investigated the polarization effect of aggregation systems, using C-X-C motif chemokine ligand 11 (CXCL11), CD68 antigen (CD68), CD80 antigen (CD80), inducible nitric oxide synthase (iNOS), interleukin-1β (IL-1β) and tumor necrosis factor-α (TNF-α) as the M1 polarized markers (**Figure S9**). As illustrated, whether aggregation, all the MNPs induced pro-inflammatory type of RAW 264.7 cell for tumor inhibition. Interestingly, the M5 and its mixture of M5&20 produced the strongest responses. Presumably, M5 with the smallest size and the highest surface-to-volume ratio could provide favorable circumstances for iron release, although M5 showed less intracellular iron content. Moreover, as shown in **Figure S10**, M5&20 exhibited the highest expression of iNOS, IL-1β and TNF-α. According to the results of flow cytometry, the IL-4 + M20&20 groups displayed distinct CD80 and CD86 fluorescent signals compared with separate IL-4 groups, which indicated M20&20 could promote macrophages polarization from M2 to M1 (**Figure 3G**). However, compared to the group without AMF (**Figure S9**), the AMF-added groups displayed the lower expression of iNOS, IL-1β and TNF-α, which may be due to the decreased activity of macrophages by magnetic heating. The above results illustrated a schematic of giant cell formation and macrophage polarization (**Figure 3H**). It is speculated that a controlled aggregation system could strengthen the tumor therapy effect not only by triggering the multinucleated giant cell formation but also by promoting M1-type polarization. As a result, the tumor microenvironment was rebuilt, showing an activated immune scenario. The formatted MGC could enhance CD4^+^ T helper cells recruitment, 41, 42 while M1 macrophages could present peptide antigens to CD4^+^ T helper cells through the major histocompatibility complex class II (MHC-II) for immune activation. 43, 44

### *In vivo* accumulation and immune activation of controlled aggregation system

**Figure 4:**
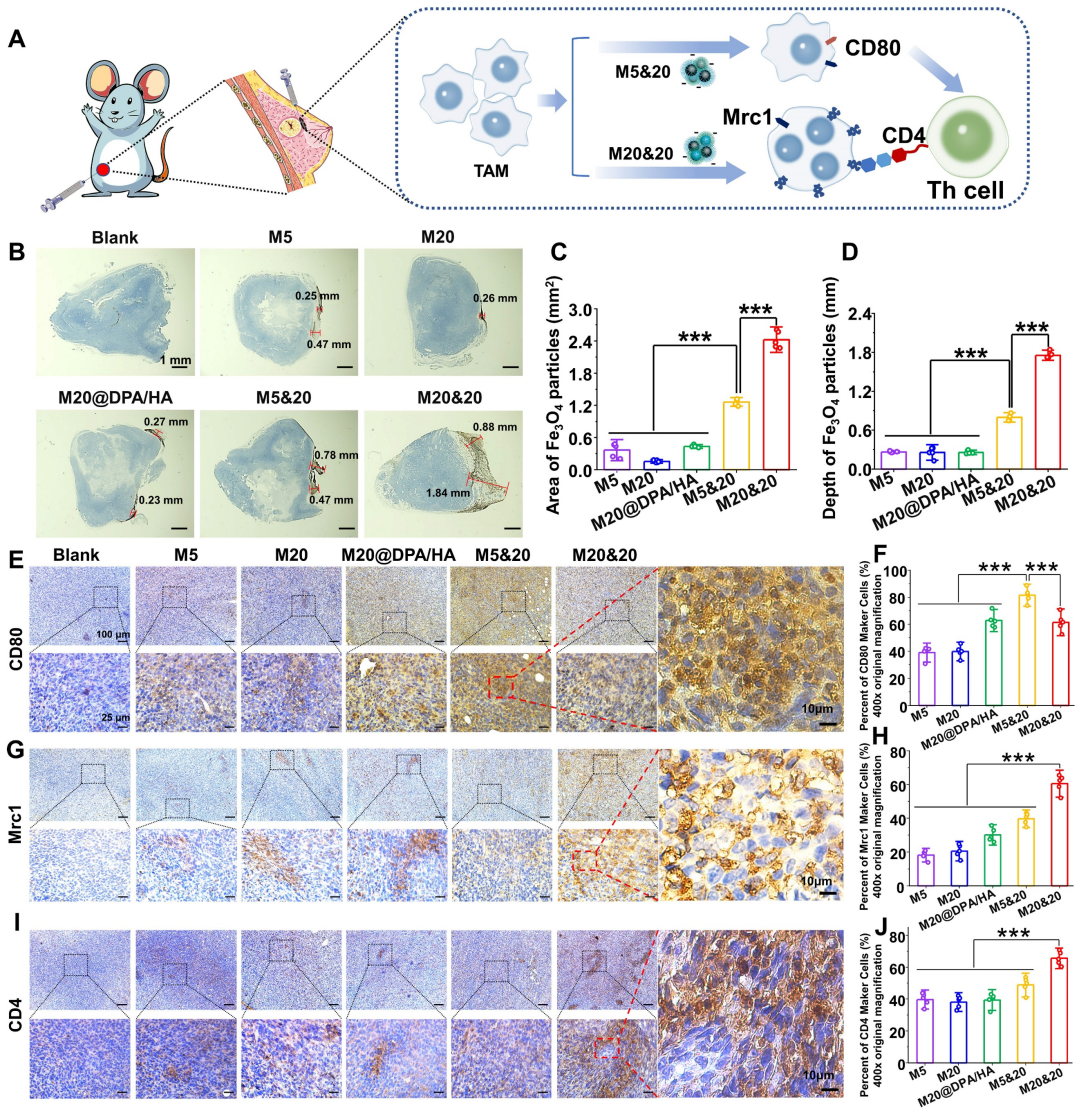
Detection of the intracellular aggregation induced permeation, retention and immune activation in vivo. (A) Schematic illustration of tumoral administration and possible immune activation process of different MNPs. (B) Permeation and retention of MNPs in tumor sections by DAB-enhanced Prussian staining in different groups. Scale bars: 1 mm. (C) Retention area (mm^2^) and (D) permeation depth (mm) of varied MNPs in tumor sections, calculated from the images of Figure 4 B. Immunohistochemical staining and positive cell percentages of CD80 (E and F), Mrc1 (G and H) and CD4 (I and J) in tumors treated with different MNPs (400 ×). Scale bars: 100, 25, and 10 μm, respectively. *** P < 0.001.

The effect of the controlled aggregation on penetration and therapy was evaluated *in vivo*. After the MNPs were injected directly into tumor tissue, the corresponding MNP accumulation and process of immune activation were investigated (**Figure 4A**). 24 h after injection, tumor sections with 3,3'-diaminobenzidine tetrahydrochloride hydrate (DAB)-enhanced Prussian staining illustrated that the mixture group (M20&20 and M5&20) harvested a better permeation and accumulation of MNPs compared to the monomer groups (M5 and M20). The M20&20 group displayed the deepest permeation of 1.84 mm and the maximum accumulation area of 2.42 mm^2^, followed by M5&20 with 0.78 mm and 1.26 mm^2^ (**Figure 4B, C and D**). Besides, the permeation and accumulation of the mixture group were far better than the monomer groups (~ 15 - 20 folds). The result confirmed our hypothesis that intracellular aggregation benefits retention of MNPs inside the tumor tissues by decreasing leakage. Moreover, according to results of ICP-OES, M20&20 groups displayed the highest Fe concentration, which indicated that M20&20 could significantly accumulate into tumor. Meanwhile, the TAM polarization and giant cell formation were also detected by the immunohistochemistry staining. The percentage of CD80 cells increased in all MNPs treated groups (**Figure 4E and F**), and the controlled aggregation groups showed higher values than others. According the immunofluorescence staining (**Figure S11**), M20&20 groups displayed the much higher mean fluorescence intensity (MFI) of F4/80 and CD86 compared with control groups, which indicated that M20&20 could promote macrophages polarization from M2 to M1 in tumor tissue. The most multinucleated giant cell formation was observed in the M20&20 group (**Figure 4G and H**), with 60.5% positive staining of Mrc1. Moreover, the CD4^+^ T helper cell accumulation also increased (**Figure 4I and J**). Meanwhile, according to immunofluorescence staining (**Figure S12**), M20&20 treated group exhibited much higher MFI of CD3/CD4 with little fluorescent signals of CD25, which indicated that M20&20 could promote the recruitment of Th cells (CD4^+^CD25^-^), but not Treg cells (CD4^+^CD25^+^) in tumor tissue. That is to say, the controlled aggregation system could also increase the intertumoral accumulation of MNPs with enhanced TAM polarization and giant cell formation* in vivo*.

### *In vivo* MHT and MRI

**Figure 5:**
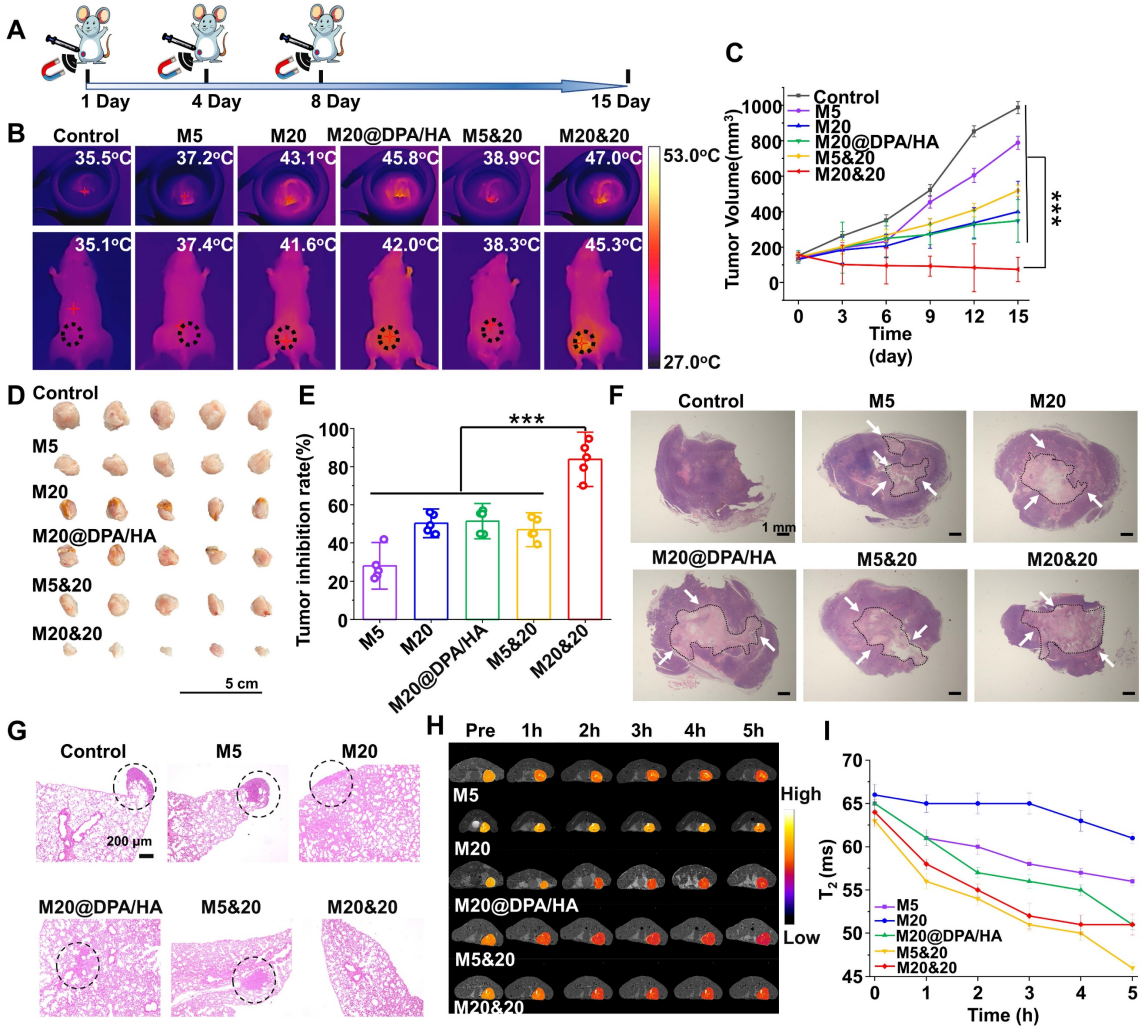
Therapeutic effect of intracellular aggregation of MNPs with MRI. (A) A schematic timeline for the administration of MNPs and magnetic hyperthermia therapy process on 4T1 tumor-baring mice. (B) Infrared thermal imaging photos of tumor-bearing mice after intratumoral injection of different MNPs followed with AMF (20 min, 15 KA/m, 300 kHz) at the day 1. (C) Tumor growth curves of mice treated with different MNPs during the treatment. (D) Morphology of tumor tissues excised from mice post 15-day treatment. (E) Tumor inhibition rate of different MNPs. (F) Tumor tissue sections of 4T1-bearing mice with H&E staining after treatment. The areas of tumor necrosis were indicated by black circles and white arrows. Scale bars: 1 mm. (G) H&E analysis of lung at day 15 post treatment. Metastatic lesions were indicated by black cycles. Scale bars: 200 μm. (H) *In vivo T*_2_-mapping images of tumor-bearing mice before and after *i.v.* injection of different MNPs at the indicated time points. (I) *T*_2_ relaxation time of various MNPs based on MRI single over time. *** P < 0.001.

Encouraged by the obtained results, the antitumor activity of the controlled aggregation system was subsequently investigated under AMF. The orthotopic 4T1-bearing mice were intratumorally injected with different MNPs at Fe concentration of 5 mg/kg, performing AMF treatment on day 1, 4, and 8 (**Figure 5A**). As monitored by an infrared thermal camera, the tumor tissue in the M20&20 treated mice quickly rose to the highest temperature of ∼ 47.0^o^C under the AMF exposure (**Figure 5B**), which was enough to kill tumor cells effectively. The change curves of tumor volume indicated that the M20&20 mixture harvested a satisfactory therapy effect under AMF, with almost no tumor growth, better than all the other groups (**Figure 5C**). The M20&20 treatment gradually decreases the tumor size, and the tumor shrunk to 52% of its original size by day15. The slight body change and H&E-stained sections of the main organs illustrated that the MNPs had an acceptable biocompatibility (**Figure S13A and S17**). Besides, routine blood and biochemical indicators related to liver and kidney function were normal, indicated that the MNPs had a good biosafety (**Table S3 and Figure S14**). The above results were expected from the highest penetration and retention of M20&20 in tumor tissue among all the groups. The morphologies and weight of tumors excised from 4T1-bearing mice were recorded (**Figure 5D and S13B**). M20&20 groups displayed minimal tumor volume, and the tumor weight of M20&20 groups was decreased by 6 folds. After calculation, M20&20 groups showed the highest inhibition rate (83.8%) (**Figure 5E**), efficiently suppressing the tumor development compared with other groups. Histopathology images of the dissected tumor tissues were collected (**Figure 5F**), and almost 68.3% tumor necrosis area could be observed in H&E images of the M20&20 treated group (**Figure S13C**), which was 5 folds higher than M5 or 3 folds higher than M20@DPA/HA. The biodistribution of the injected magnetothermal nanoparticles was evaluated *in vivo*. According to results of ICP-OES (**Figure S15**), compared with PBS and M5&20 groups, M20&20 groups displayed the highest Fe concentration in liver, spleen, and tumor. In addition, compared with PBS and M20&20 groups, M5&20 groups displayed the highest Fe concentration in kidney. The results indicated that M20&20 could significantly accumulated in tumor (**Figure S16**). Moreover, the aggregation of M20&20 could inhibit pulmonary metastases of breast tumor cells (**Figure 5G and S18**). The metastasis foci were observed in control, M5, M20, M20@DPA/HA and M5&20 groups but not in M20&20 groups. That is mainly because the mixture of M20&20 not only performed favorable MHT effect but also promoted immune activation through intracellular aggregation.

Meanwhile, the MRI property of MNP aggregations was detected under 7.0 T MR scanner. As shown in **Figure 5H** and** S19A**, M5&20 and M20&20 were more sensitive as *T_2_* contrast agents than M5, M20, and M20@DPA/HA groups in the breast tumor model. According to curves of *T*_2_ relaxation time (**Figure 5I**) and signal-to-noise ratio (SNR) over post-injection time (**Figure S19B**), the M5&20 groups had the lowest *T*_2_ relaxation time and SNR value than other groups after injected 5 h, which is consistent with the in vitro study (**Figure 1**).

## Conclusion

In summary, we developed a controlled intracellular aggregation system. Under the acidic condition, the system displayed an aggregation behavior while maintaining stability in the neutral condition. It exhibited improvement of magneto-thermal conversion efficiency and *T*_2_ relaxivity, acquired the most efficient ROS generation and tumor cell killing by enhanced intracellular accumulation, as well as induced M1 polarization and MGC formation of TAM, which further facilitates tumor inhibition by immune activation. As a result, this controlled aggregation system harvested a significantly enhanced MHT effect with barely tumor growth and metastasis by increased permeation and intracellular retention inside tumor tissues and immune activation. In our opinion, controlling the intracellular aggregation of MNPs is a promising strategy to improve MHT and MRI in cancer therapy.

## Materials and Methods

### Experimental materials

3-(3,4-Dihydroxyphenyl) propionic acid was provided by Alfa Aesar (USA). *N,N*-diisopropylethylamine (DIEA), ethylene glycol, phosphorus trichloride, o-(benzotriazol-1-yl)-*N,N,N′,N′*-tetramethyluronium tetrafluoroborate (TBTU), diethylene glycol, sodium periodate (NaIO_4_) and tert-butyl carbazate were obtained from Adamas-beta (China). N-[(3-trimethoxysilyl) propyl] ethylenediamine triacetic acid trisodium salt, 40 wt.% solutions in H_2_O (TEAT) and trifluoroacetic acid (TFA) were purchased from J&K (China). Hyaluronic acid (HA, 6 kDa) was obtained from Bloomage Biotechnology Corporation Limited (China). Iron (III) acetylacetonate (Fe(acac)_3_) was purchased from Aldrich (USA). Ferric chloride hexahydrate (FeCl_3_ ·6H_2_O) and ferrous chloride tetrahydrate (FeCl_2_ ·4H_2_O) were obtained from Adamas-beta (China). 3,3'-diaminobenzidine tetrahydrochloride hydrate (DAB) was purchased from TCI (Japan). All the other chemicals were purchased from Kelong Chemical Reagent Corporation (China) and used without further purification.

Mouse macrophage cells (RAW264.7) and breast cancer cells (4T1) were obtained from the Chinese Academy of Science Cell Bank for Type Culture Collection (China), which were characterized by cytogenetic karyotyping and short tandem repeat profiling and passed the detection of Mycoplasma. (RPMI)-1640 medium, phosphate buffer saline (PBS), and fetal bovine serum (FBS) were purchased from Hyclone (USA). The Calcein/PI Live/Dead Viability/Cytotoxicity assay kit (C2015M) and propidium iodide (PI, ST511) assay kit were purchased from Beyotime (China). The reactive oxygen species (ROS) assay kit (2,7-dichlorofluorescein diacetate, DCFH-DA) was obtained from Beyotime (China). Primary antibodies against CD80, Mrc1, and CD4 were obtained from Boster (China). The primers for quantitative real-time polymerase chain reaction (Q-PCR) included TNF-α, IL-1β, iNOS, SR-A, CXCL11, CD68, CD80, H2-Eb1 were purchased from Tsing Ke Biological Technology (China). Trizol was obtained from Thermo (USA). iScript™ cDNA synthesized kit and SosoFast EvaGreen Supermix were purchased from Bio-Rad (USA). Triton X-100 was purchased from Biosharp Ltd. (China). 4′,6-diamidino-2-phenylindole (DAPI), and Prussian blue assay kit were purchased from Solarbio Technology Ltd. (China). Trypan blue was purchased from Sigma (USA). CD80 (PE hamster anti-mouse) and CD86 (APC rat anti-mmouse) were purchased from BD Biosciences. F4/80 (GB11027), CD3 (GB111337), CD4 (GB13064-2), CD25 (GB11584) and CD86 (GB13585) were purchased from Servicebio (China).

### Synthesis and characterization of negatively charged MNPs

MNPs of 5 nm were prepared according to previous literature. 25 Fe(acac)_3_ (0.4 g, 0.001 mol) and diethylene glycol (20 mL, 0.210 mol) were added into three-necked flask under nitrogen. The solution was heated at 140^o^C for 1 h and kept at 200^o^C for 5 h. After cooling to room temperature, ethanol/ether (v/v = 1/8) was added to the reaction solution for precipitation. After centrifugation and washing with ethanol/ether, the obtained MNPs (50 mg) were suspended in deionized water (50 mL). TEAT (0.5 mL) was slowly added, followed by stirring at 30^o^C for 12 h. The reaction solution was dialyzed against ultrapure water (MWCO 3.5 kDa) for 2 days, and centrifuged at 12,000 rpm/min for 10 min to obtain the final negatively charged MNPs (M5).

MNPs with a size of 20 nm were fabricated by the general chemical coprecipitation method. 24 FeCl_3_·6H_2_O (1.0 g, 4 mmol) and FeCl_2_·4H_2_O (0.4 g, 2 mmol) were firstly dissolved in deionized water (50 mL) and slowly dropped into sodium hydroxide solution (50 mL, 1.5 mol/L) with vigorous mechanical stirring for 4 h. During the reaction, some dark precipitates formed. They were collected by magnetic separation and washed with ultrapure water. The obtained MNPs (50 mg) were dispersed into toluene under nitrogen, and TEAT (0.5 mL) was slowly added with stirring at 30^o^C for 12 h. After the following dialysis and centrifugation as the treatment of M5, the negatively charged MNPs (M20) were obtained.

The crystal structure of MNPs was detected by powder X-ray diffraction (XRD) (X'Pert PRO, Holland) with an angle range of 20^o^ ~ 90^o^ and scanning speed of 0.1 ^o^/s. Size distribution and zeta potentials were measured by the Malvern instrument Zetasizer Nano system (Zetasizer NanoZS, Malvern, UK). The morphology of particles was observed by TEM (Tecnai G2 F20 S-TWIN, USA). The surface functional group of MNPs was detected by FTIR spectra (4000 - 400 cm^-1^, Nicolet6700, Thermo). The thermogravimetric curve was measured by a thermal gravimetric analyzer (TGA, Mettler Toledo, Switzerland), with a temperature range of 35 ~ 1000^o^C and a heating rate of 10^o^C/min.

### Synthesis and characterization of MNPs with pH-responsive charge reversal properties (M20@DPA/HA)

To a solution of 3-(3,4-Dihydroxyphenyl) propionic acid (2.0 g, 10 mmol) in anhydrous acetone (100 mL) at 0^o^C, phosphorus trichloride (0.8 mL, 0.008 mmol) was dropwise added with stirring in an ice bath for 6 h. After the removal of organic solvent by distillation, the residue was redissolved in diethyl ether and washed with deionized water. After the collection of the organic layer and removal of organic solvents using vacuum rotary evaporation, a white powder named DPAA was obtained, with a yield of approximately 36%.

Followingly, DPAA (0.5 g, 2 mmol), tert-butyl carbazate (0.3 g, 2 mmol), an appropriate amount of TBTU, and DIEA were dissolved in dimethylformamide with stirring overnight at 30^o^C under nitrogen. After the removal of the organic solvent, the resultant residue was purified by silica column chromatography (dichloromethane/methanol, v/v = 10/1), to obtain a light yellowish solid (BOC-DPAA) with a yield of 70%. ESI-MS (LC-MS) m/z 335.15 [M - H] (calcd for C_17_H_24_N_2_O_5_, 336.17); ^1^H NMR (400 MHz, CDCl_3_) δ 6.59 (s, 3H), 2.96 (s, 2H), 2.49 (s, 2H), 1.64 (s, 6H), 1.44 (s, 9H).

The above-obtained BOC-DPAA (1.0 g, 3 mmol) was dissolved in dichloromethane (3 mL) with TFA (2 mL) for a continuous 24 h-stirring at room temperature. After the removal of organic solvent, the obtained product (DPA) was precipitated by diethyl ether and dried in vacuum (0.5 g, 76% yield). HRMS m/z 195.0805 [M - H] (calcd for C_9_H_12_N_2_O_3_, 196.0848); ^1^H NMR (400 MHz, D_2_O) δ 6.76 (s, 1H), 6.70 (s, 1H), 6.62 (s, 1H), 2.76 (s, 2H), 2.52 (s, 2H).

The hyaluronic acid with aldehyde groups (HA-CHO) was synthesized using sodium periodate (NaIO_4_) oxidization. 31, 32, 45, 46 Briefly, HA (5.0 g, 12 mmol) was dissolved in deionized water (50 mL), followed by the addition of NaIO_4_ (5.0 g, 23 mmol). The reaction solution was stirred overnight in dark, and terminated by 5 mL of ethylene glycol. The excess reactant and by-product were removed by dialysis (MWCO 3.5 kDa) against deionized water for 2 days. After freeze-drying, a white flocculent solid (HA-CHO) was collected, with a yield of 98% and structure confirmation by ^1^H NMR and FTIR.

MNPs of 20 nm (100 mg) and DPA (300 mg) were dispersed in methanol (4 mL) with stirring for a 6 h reaction at 50^o^C under argon. The precipitates (M20@DPA, 50 mg) were collected by magnetic separation and washed with methanol. They were then mixed with HA (300 mg, 0.73 mmol) in PBS solution (50 mL, pH 7.4) overnight. The final product (M20@DPA/HA) was obtained by magnetic separation, When the reaction of M20@DPA and HA-CHO was completed, the obtained pH-responsive charge reversal MNPs (M20@DPA/HA) was collected by magnetic separation and followed by washing with ultrapure water for three times.

M5, M20 and M20@DPA/HA were vacuum-dried for 24 hours, powder of various MNPs were added into vibrating sample magnetometer under magnetic field of 20000 Oe (8600 VSM, Lake Shore, USA) to detect the hysteresis curves of different MNPs.

The structures of midbodies were characterized by nuclear magnetic resonance (NMR spectrometry (Bruker AV II-400 MHz, Switzerland) as well as liquid chromatograph mass spectrometer (LCMS-IT-TOF, Shimadzu, Japan; or LC-MS, TSQ Quantum U1tra, Thermo Fisher Scientific USA).

### pH-responsive aggregation of MNPs (M5&20 and M20&20)

M5 (or M20) was mixed with M20@DPA/HA by gently blending at equal volume with the same Fe concentration (0.5 mg/mL) to obtain the mixture solution of M5&20 (or M20&20). Next, they were dispersed in PBS with pH 7.4, 6.8, or 5.5 for aggregation exploration. Samples of mixed MNPs in pH 5.5 were expected to aggregate, named A-M5&20 or A-M20&20. Changes in size distribution and zeta potential were detected by the Malvern instrument (Zetasizer Nano ZS, Malvern, UK), and the morphologies were observed by TEM (Tecnai G2 F20 S-TWIN, USA).

### *In vitro* magneto-thermal conversion capacity and MRI evaluation of varied MNPs

Magneto-thermal conversion capacity of varied MNPs (M5, M20, M20@DPA/HA, A-M5&20, and A-M20&20) was evaluated at a series of Fe concentration (2, 1, and 0.5 mg/mL) under alternating magnetic field (AMF, SuperMag Technology, China) with the power of 15 KA/m (300 kHz). PBS and DMEM were put into the magnetic heat coil with AMF (15 KA/m, 300 kHz, SuperMag Technology, China) for 20 min. The SAR was calculated as follows:



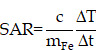



where *c* is the heat capacity of medium (water, 4.2 × 10^3^ J/kg·^o^C), *ΔT* is the temperature variation, *Δt* is the change of time, *ΔT/Δt* is the temperature increasing rate,* m_Fe_* is the weight fraction of Fe in the medium. After the addition of AMF, infrared thermal imaging was conducted by a far-infrared thermometer (HIKVISION, H13, China).

MNPs were embedded in agarose gel (1%) at different Fe concentrations (0.03, 0.06, 0.10, 0.15 and 0.25 mM) and subjected to the MRI scan. The MRI was conducted on a 7.0 T preclinical MRI system (NOVA 7.0 T, Time Medical Ltd.), and obtained using spin echo sequence (repetition time (TR), 5000 ms; echo time (TE), 6.9 - 500 ms; matrix, 384 × 224; field of view (FOV), 250 × 190 mm^2^; slice thickness, 2.0 mm. The *T*_2_ relaxation times were calculated by fitting these multiple spin echo images.

### Cellular uptake of MNPs

RAW264.7 and 4T1 cells were cultured in RPMI-1640 medium supplemented with 10% FBS in 5% CO_2_ at 37^o^C with saturated humidity. Cells were seeded into a 24-well plate at a density of 2.5 × 10^5^ cells/well and cultured for 24 h. Afterward, various MNPs (M5, M20, M20@DPA, M20@DPA/HA, A-M5&20, A-M20&20, M5&20, and M20&20) were added per well at a final concentration of 5 μg Fe/mL followed with a 24 h co-incubation. After removal of the medium, cells were washed with PBS and fixed with 4% paraformaldehyde for 30 min. Cellular uptake of varied MNPs was detected by Prussian blue staining and ICP-OES analysis (5100 SVDV, Agilent, USA).

### Intracellular aggregation formation of varied MNPs

4T1 cells were firstly seeded in 60 mm plates with a 24 h-incubation. Various MNPs (M5, M20, M20@DPA/HA, M5&20, and M20&20) were added to the wells at a final concentration of 5 μg/mL. 24 h later, all groups were successively prefixed with 3% glutaraldehyde, postfixed with 1% osmium tetroxide, dehydrated in series acetone, infiltrated in upon (Fullam Epox 812), and embedded after removing the treatment medium. The ultrathin sections were cut with a diamond knife and stained with uranyl acetate and lead citrate for TEM observation (JEM-1400-FLASH).

### Tumor cell killing and ROS assay in cells treated with varied MNPs

Cells were cultured, seeded, and co-incubated with varied MNPs as described in Section 2.6. All groups (including M5, M20, M20@DPA, M20@DPA/HA, A-M5&20, A-M20&20, M5&20, and M20&20) were then treated with AMF (300 kHz, 15 KA) for 20 min, followed by another 30 min-incubation.

*Live and dead analysis* Cells were stained with calcein acetoxymethyl ester (calcein-AM, 2 μmol/L) and PI (4.5 μmol/L) solutions for 30 min according to the instruction. After removal of the staining solution, cells were washed with PBS before observation using a fluorescent microscope (Olympus X70, Japan).

*PI staining of apoptosis* Cells was digested with trypsin and suspended in PBS (0.5 mL). They were then incubated with PI solution (10 μg/μL) for 30 min and analyzed by flow cytometry (BD FACSCelesta, USA) using excitation of 535 nm and emission of 617 nm.

*Dead cell ratio* Trypan blue solution was added into the cell suspension at a final concentration of 0.4‰. And then, the dead cell ratio was measured by cell counting apparatus (JSY-SC-031, BodBoge, China).

*ROS detection* DCFH-DA with 1‰ concentration in the fresh medium was added to the attached cells followed by a 30 min-incubation. After a thorough wash, the stained cells were observed using an inverted fluorescence microscope (Olympus X70, Japan), and the MFI was quantitively analyzed using the ImageJ software.

### Polarization and giant cells formation of macrophages

RAW264.7 cells were seeded into a 6-well plate at a density of 1.2 × 10^6^ cells per well and cultured for 24 h. After being treated with various MNPs, the medium was replaced with fresh ones. Macrophages were stained with Prussian blue and analyzed by ICP-OES. The mononucleated and multinucleated cells (≥ 3) were counted during the observation using an inverted microscope. Next, the average number and percentage acreage (%) of multinucleated cells were calculated or measured by ImageJ software.

For analysis of gene expression related to polarization and giant cell formation of macrophage, RAW cells were co-incubated with various MNPs at a final concentration of 5 μg/mL for 24 h. And then, they were subjected to the reverse transcription Q-PCR. In addition, RAW264.7 cells were pretreated with M2 polarizing factor IL-4 (20 ng/mL) for 24 h, and then treated with various MNPs at a final concentration of 5 μg/mL for 24 h. After then, various MNPs were treated with AMF (15 KA/m) for 20 min and cells were subjected to the reverse transcription Q-PCR. In brief, total RNA was isolated from the treated cells with Trizol reagent according to the manufacturer's instructions. After the complementary DNA was transcribed using an iScript™ cDNA synthesized kit, Q-PCR was performed with SosoFast EvaGreen Supermix in a 7300 Real-Time PCR System (Applied Biosystems, USA), with designed primer sequences (**Table S1**). The expression of specific genes, including TNF-α, IL-1β, iNOS, Mrc1, SR-A, CXCL11, CD68, CD80, H2-Eb1 were evaluated, with standardization by the expression of β-actin. For cytoskeleton staining, the MNPs treated macrophages were fixed with 4% paraformaldehyde for 10 min and thoroughly washed with PBS. Afterward, 0.5% Triton X-100 was added for permeabilization, followed by successive addition of rhodamine phalloidin (1: 100) and DAPI for 30- and 10-min incubation, respectively. Finally, the filamentous actin (F‐actin, red) and the nuclei (blue) of the giant cells were observed by a confocal laser scanning microscope (Zeiss, Germany).

Flow cytometry was employed to evaluate the CD80 (PE hamster anti-mouse) and CD86 (APC rat anti-mouse) expression. RAW264.7 cells were pretreated with M2 polarizing factor IL-4 (20 ng/mL) for 24 h, followed by co-incubation with M20&20 for another 24 h. After that, 1×10^6^ cells were digested and collected. CD80 and CD86 antibody (1 μg/mL) were then added into 100 μL of cell suspension, and incubated at room temperature for 30 min. After washing by PBS triply, cells were collected and subjected to the FACS aria flow cytometer (BD Biosciences) with FlowJo software.

### Animal experiment

Female BALB/c mice (4 - 6 weeks old, ~ 20 g) were purchased from Gempharmatech Co., Ltd. (China). [License number: SCXK (Chuan) 2020-034]. All animal experimental procedures were performed in accordance with the Guidelines for Care and Use of Laboratory Animals of West China Hospital of Sichuan University and approved by the Biomedical Research Ethics Committee of West China Hospital of Sichuan University (20220307037). The 4T1 mouse mammary tumor model was established by orthotopic injection of 4T1 cells (1 × 10^6^ cells in 50 μL PBS) into the left fourth mammary fat pad of an age-matched female mouse. When the tumors reach ~ 100 mm^3^, the following experiments were performed. In the MHT experiment, the mice were divided into 6 experimental groups (Control, M5, M20, M20@DPA/HA, M5&20, and M20&20), with 5 mice in each group. When investigating the retention of MNPs, the mice were divided into 6 experimental groups (Control, M5, M20, M20@DPA/HA, M5&20, and M20&20), with 3 mice each.

*Enhanced MRI scan* MNPs (M5, M20, M20@DPA/HA, M5&20, and M20&20) were injected into tumor-bearing mice via tail vein (2.8 mg Fe/kg), respectively. *T*_2_-weighted images and *T*_2_-mapping images were acquired using a mouse coil-equipped 7.0 T MR scanner with a fast spin echo sequence (TR, 3500 ms; TE, 30 ms; FOV, 45 × 45 mm^2^; matrix, 256 × 256; slice thickness, 1.0 mm) before and after the injection at the indicated time points. The relative signal-to-noise ratio (SNR) was also calculated.

*In vivo fate and immune activation* MNPs (including M5, M20, M20@DPA/HA, M5&20, and M20&20) (5 mg Fe/kg) were peritumorally (not intratumorally) injected. Various MNPs were injected uniformly into the surface layer of the tumor near the breast, the location was consistently in the area of subcutaneous and just above the tumor to explore the penetration, aggregation, and retention of particles. Tumor tissues were dissected 24 h after injection and were orderly fixed in 4% neutral buffered formaldehyde, dehydrated in gradient alcohol, and embedded by paraffin. Tumors were then sliced (5 μm), and treated with 2% potassium ferrous hydride and 2% hydrochloric acid for 30 min. Slides were stained with DAB color droplets and rinsed with PBS solution. After 4T1 mouse mammary tumor model was established, PBS, M5&20 and M20&20 were intravenously injected. After 24 h, tumors and main organs (heart, liver, spleen, lung, and kidney) were dissected and lysed with nitric acid for 48 h. Then the Fe concentration of tumor was detected by ICP-OES. H&E staining of lung treated with various MNPs was collected and the number of lung metastases was counted. The immunohistochemistry staining was performed with primary antibodies of CD80 (anti-mouse, 1: 200), Mrc1 (anti-mouse, 1: 200), and CD4 (anti-mouse, 1: 200) for immune activation detection. After incubation with antibodies overnight at 4^o^C and washing with PBS, the tissue slides were followingly incubated with horseradish peroxidase-conjugated goat anti-rabbit secondary antibodies (1: 2000) at room temperature for 40 min, and finally visualized with 0.2% diaminobenzidine tetrahydrochloride and hydrogen peroxide. Ten visual fields at 400× the original magnification of each section was taken, and the percentage of CD80, Mrc1, and CD4 positive cells per field was calculated to assess the average value in every group.

The tissue sections were firstly subjected to antigen repair, and then the primary antibody F4/80 (1: 200, GB11027, Servicebio) and CD86 (1: 1000, GB13585, Servicebio) were added and incubated for 24 h. The corresponding secondary antibody was added and incubated for 50 min. DAPI staining (0.5 μg/mL) was used to visualize nuclei. Antifade Mounting Medium was utilized to coverslip all sections.

The primary antibody CD3 (1: 200, GB111337, Servicebio), CD4 (1: 2000, GB13064-2, Servicebio) and CD25 (1: 3000, GB11584, Servicebio) were added and incubated for 24 h. The corresponding secondary antibody was added and incubated for 50 min. DAPI staining (0.5 μg/mL) was used to visualize nuclei. Antifade Mounting Medium was utilized to coverslip all sections. All sections were observed using a fluorescence microscope (NIKON ECLIPSE C1, Japan), and the MFI was quantitively analyzed using the ImageJ software.

*MHT of varied MNPs* After anaesthetization, mice were intratumorally injected with MNPs at Fe concentration of 5 mg/kg on Day 1, 4, and 8, and treated with AMF (15 KA/m, 300 kHz) for 20 min. Infrared thermal imaging was conducted by a far-infrared thermometer (HIKVISION, H13, China). A vernier caliper was used to measure the length (L), width (W), and height (H) of the tumor, for volume calculation using V (mm^3^) = 1/2 × L × W × H. All mice were sacrificed after 15 day-treatment. Tumor tissues and other organs were manually dissected, they were orderly fixed in 4% neutral buffered formaldehyde, dehydrated in gradient alcohol, and embedded by paraffin. After tissues were sliced (5 μm), they were deparaffinized and hydrated for further hematoxylin and eosin (H&E) staining.

After 4T1 mouse mammary tumor model was established, PBS, M5&20 and M20&20 were injected into mice by intravenous injection. After 24 h, the blood was collected and detected by auto hematology analyzer (BC-2800 Vet, Mindray, China) and chemistry analyzer (BS-240 Vet, Mindray, China). Alanine aminotransferase (ALT), aspartate aminotransferase (AST), alkaline phosphatase (ALP) and albumin (ALB) were related to liver function. Carbamide (UREA), creatinine (CREA), uric acid (UA) and glucose (Glu) were related to kidney function.

### Statistical analysis

All data are expressed as means standard deviations (± S.D.). The differences among various groups were analyzed by t-tests. Values of p < 0.05 (*) were considered to be statistically significant and P < 0.01 (**) and P < 0.001 (***) were considered highly statistically significant.

## Supplementary Material

Supplementary figures and tables.

## Abbreviations

4T1: breast cancer cells
AMF: alternating magnetic field
calcein-AM: calcein acetoxymethyl ester
CD68: CD68 antigen
CD80: CD80 antigen
CXCL11: C-X-C motif chemokine ligand 11
DAB: 3,3'-diaminobenzidine tetrahydrochloride hydrate
DAPI: 4′,6-diamidino-2-phenylindole
DIEA: *N,N*-diisopropylethylamine
FBS: fetal bovine serum
Fe(acac)_3_: iron (III) acetylacetonate
FeCl_2_ ·4H_2_O: ferrous chloride tetrahydrate
FeCl_3_ ·6H_2_O: ferric chloride hexahydrate
FTIR: Fourier transform infrared
FOV: field of view
H2-Eb1: histocompatibility 2, class II antigen E beta
HA: hyaluronic acid
ICP-OES: inductively coupled plasma optical emission spectrometry
IL-1β: interleukin-1β
iNOS: inducible nitric oxide synthase
MFI: mean fluorescence intensity
MGC: multinucleated giant cells
MHC-II: major histocompatibility complex class II
MHT: magnetic hyperthermia therapy
MNPs: magnetic nanoparticles
Mrc1: mannose receptor C-type 1
MRI: magnetic resonance imaging
NaIO_4_: sodium periodate
PBS: phosphate buffer saline
PI: propidium iodide
Q-PCR: quantitative real-time polymerase chain reaction
*r*_2_: *T*_2_ relaxivity
RAW264.7: mouse macrophage cells
ROS: reactive oxygen species
SAR: specific absorption rate
SNR: signal-to-noise ratio
SR-A: class A scavenger receptor
TAM: tumor-associated macrophages
TBTU: o-(benzotriazol-1-yl)-*N,N,N′,N′*-tetramethyluronium tetrafluoroborate
TE: echo time
TEAT: N-[(3-trimethoxysilyl) propyl] ethylenediamine triacetic acid trisodium salt, 40 wt.% solutions in H_2_O
TEM: transmission electron microscopy
TFA: trifluoroacetic acid
XRD: X-ray diffraction
