# Supplementary material for: Controlled intracellular aggregation of magnetic particles improves permeation and retention for magnetic hyperthermia promotion and immune activation
Source: Theranostics. 2023 Feb 27;13(4):1454–69. doi: 10.7150/thno.80821 (PMC10008738; doi:10.7150/thno.80821)
Supplement: Supplementary file 1 — Supplementary figures and tables. [file thnov13p1454s1.pdf]

Supporting Information

Controlled intracellular aggregation of magnetic particles improves permeation and retention for magnetic hyperthermia promotion and immune activation

Ao Hu[1, 2], Yiyao Pu[1, 2], Na Xu[1, 2], Zhongyuan Cai[1, 2], Ran Sun[3], Shengxiang Fu[1, 2], Rongrong Jin*[, 1, 2], Yingkun Guo[3], Hua Ai[1, 2], Yu Nie*[, 1, 2], and Xintao Shuai[4]

[1]National Engineering Research Center for Biomaterials, Sichuan University, Chengdu, 610064, P. R. China

[2]College of Biomedical Engineering, Sichuan University, Chengdu, 610065, P. R. China

[3]Development and Related Diseases of Women and Children Key Laboratory of Sichuan Province, West China Second University Hospital, Sichuan University, Chengdu 610041, P. R. China

[4]Nanomedicine Research Center, The Third Affiliated Hospital of Sun Yat-sen University, Guangzhou 510630, China

Table 1. Primer nucleic acid sequence for detection giant cells formation and macrophages polarization related gene.

Gene Name	Primer sequence（5‘-3’）
Mrc1-Forward	ACGAGCAGGTGCAGTTTACA
Mrc1-Reverse	TCAGGAGTTGTTGTGGGCTC
SR-A-Forward	CCAAACGCACTCCCCTTACT
SR-A-Reverse	CCACACCAGTAGCAGGACAG
CXCL11-Forward	GAACAGGAAGGTCACAGCCATA
CXCL11-Reverse	CTCTGCCATTTTGACGGCTTT
CD68-Forward	GGGGCTCTTGGGAACTACAC
CD68-Reverse	GTACCGTCACAACCTCCCTG
CD80-Forward	TTCACCTGGGAAAAACCCCC
CD80-Reverse	CCCGAAGGTAAGGCTGTTGT
H2-Eb1-Forward	ATAAATTCCTTGTGCGGCGG
H2-Eb1-Reverse	CCAGTCTCCATTTCGGACCA
TNF-α-Forward	CTGAACTTCGGGGTGATC
TNF-α-Reverse	TCCTCCACTTGGTGGTTT
iNOS-Forward	CACGGACGAGACGGATAG
iNOS -Reverse	CACTGACACTTCGCACAAA
IL-1β-Forward	AGCACCTTCTTTTCCTTC
IL-1β-Reverse	TGCCGTCTTTCATTACAC

Table S2. Calculated specific absorption rate (SAR) (W/g) and T_2 relaxivity (r_2) ($mM^{-1}s^{-1}$) values of various MNPs.

	SAR (W/g)	r_2 ($mM^{-1}s^{-1}$)
M5	41.8	48.3
M20	560.1	279.0
M20&DPA/HA	547.6	232.1
M5&20	413.8	579.6
M20&20	844.4	465.1

Table S3. Blood routine and biochemical indicators of mice treated with different MNPs.

	White blood cell count (× 10^9/L)	Lymphocytes (%)	Red blood cell count (× 10^{12}/L)	Hemoglobin (g/L)
Control	1.6 ± 0.2	76.8 ± 2.8	7.32 ± 0.4	143 ± 13
M5&20	1.0 ± 0.4	71.6 ± 2.7	7.03 ± 0.3	114 ± 15
M20&20	1.8 ± 0.5	86.0 ± 2.3	7.48 ± 0.3	140 ± 10

Continued **Table S3.** Blood routine and biochemical indicators of mice treated with different MNPs.

	Hematocrit (%)	Mean corpuscular hemoglobin (pg)	Platelet count (10^9/L)
Control	34.8 ± 3.1	18.5 ± 1.2	590 ± 35
M5&20	44.4 ± 4.2	16.9 ± 1.5	1061± 54
M20&20	35.9 ± 3.2	18.9 ± 1.4	499 ± 42

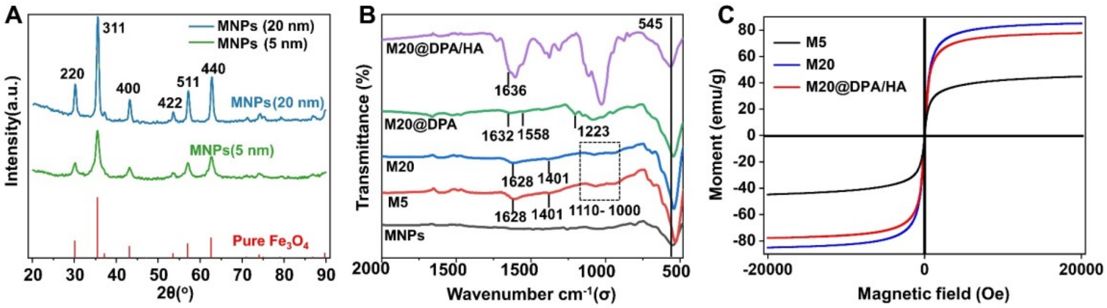

Figure S1. Characterization of various MNPs. (A) XRD spectra of MNPs with 5 nm and 20 nm. (B) FTIR spectra of various MNPs (MNPs, M5, M20, M20@DPA and M20@DPA/HA). (C) Hysteresis loops of M5, M20 and M20@DPA/HA at 20000 Oe.

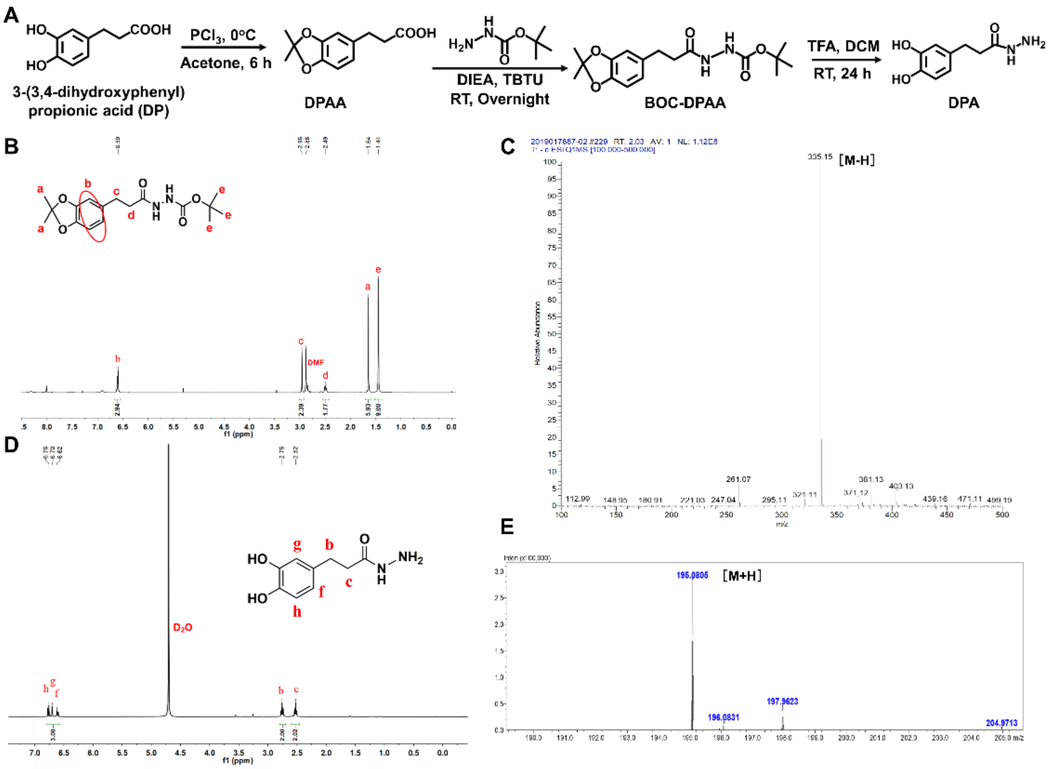

Figure S2. Characterization of BOC-DPAA and DPA. (A) Synthesis process of BOC-DPAA and DPA. (B) ^{1}H NMR and (C) mass spectrum of BOC-DPAA. (D) ^{1}H NMR and (E) mass spectrum of DPA.

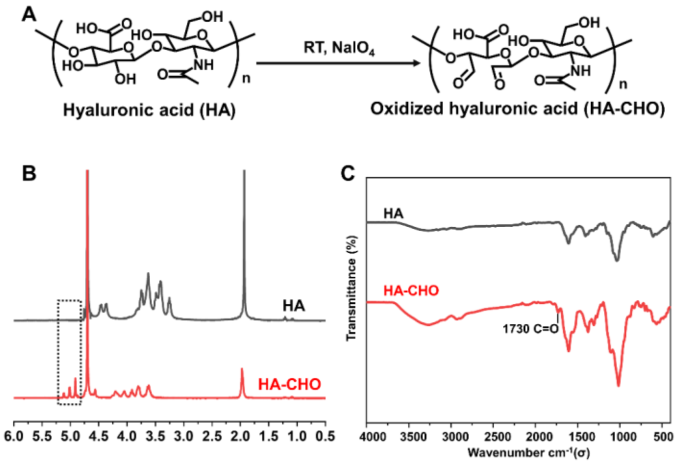

Figure S3. Characterization of HA and HA-CHO. (A) Synthesis process of HA-CHO. (B) ^{1}H NMR and (C) FTIR spectra of HA and HA-CHO.

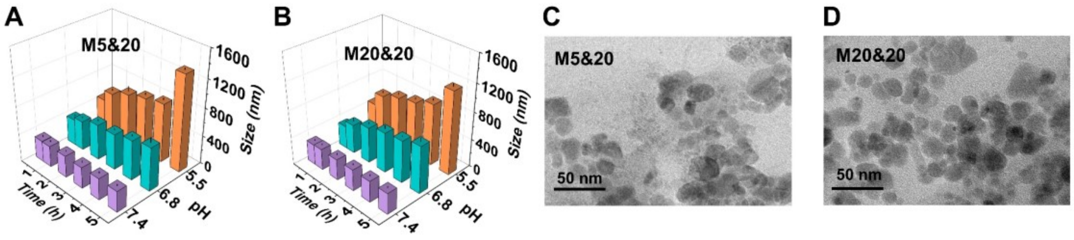

Figure S4. Characterization of different individual MNPs and pH-responsive aggregation. Size distribution of (A) M5&20 and (B) M20&20 incubated in different pH conditions for 5 h. TEM images of (C) M5&20 and (D) M20&20 incubated in pH 7.4 for 5 h. Scale bars: 50 nm.

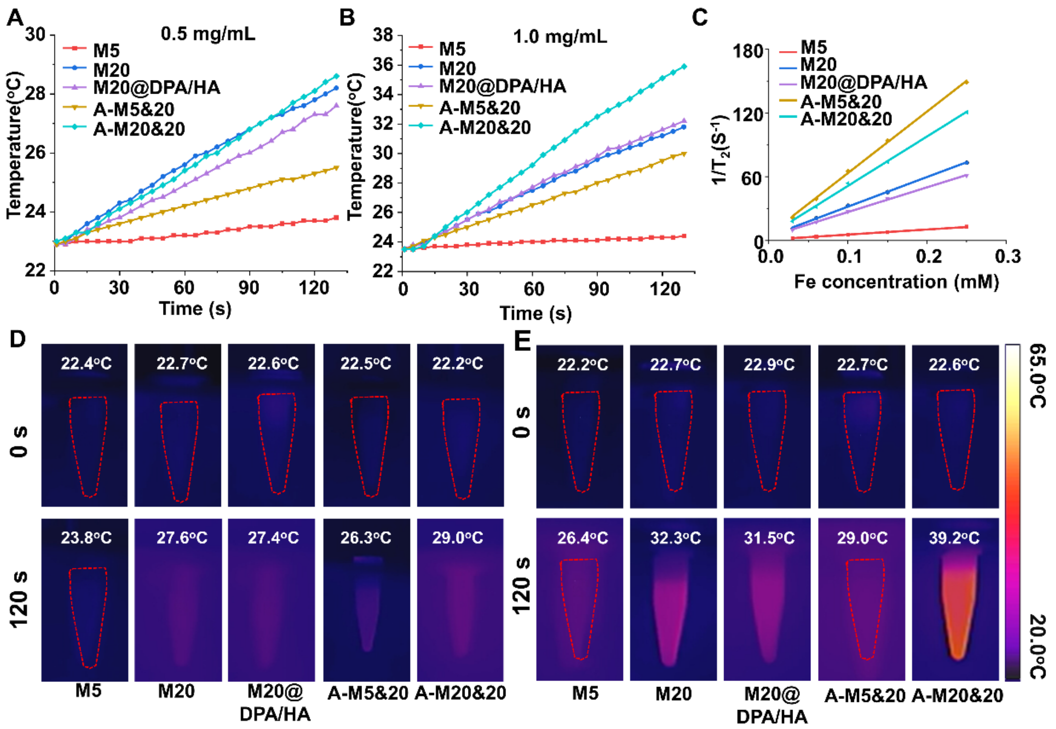

Figure S5. Magneto-thermal conversion efficiency and MRI performance of varied MNPs. Temperature change curves over time of individual MNPs and its aggregation under AMF (15 KA/m, 300 kHz) with (A) 0.5 mg Fe/mL and (B) 1.0 mg Fe/mL. (C) T_2 relaxation rate ($1/T_2$, s^{-1}) as a function of Fe concentration (mM) for different MNPs and aggregations

under a 7.0 T magnetic field. Infrared thermal imaging photos of MNPs and aggregations under AMF for 120 s with (D) 0.5 mg Fe/mL and (E) 1.0 mg Fe/mL, respectively.

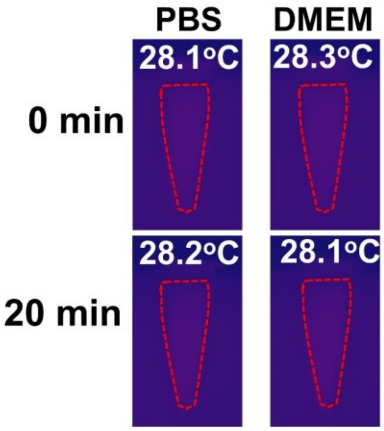

Figure S6. Infrared thermography of PBS and DMEM with AMF (15 KA/m, 300 kHz) for 20 min.

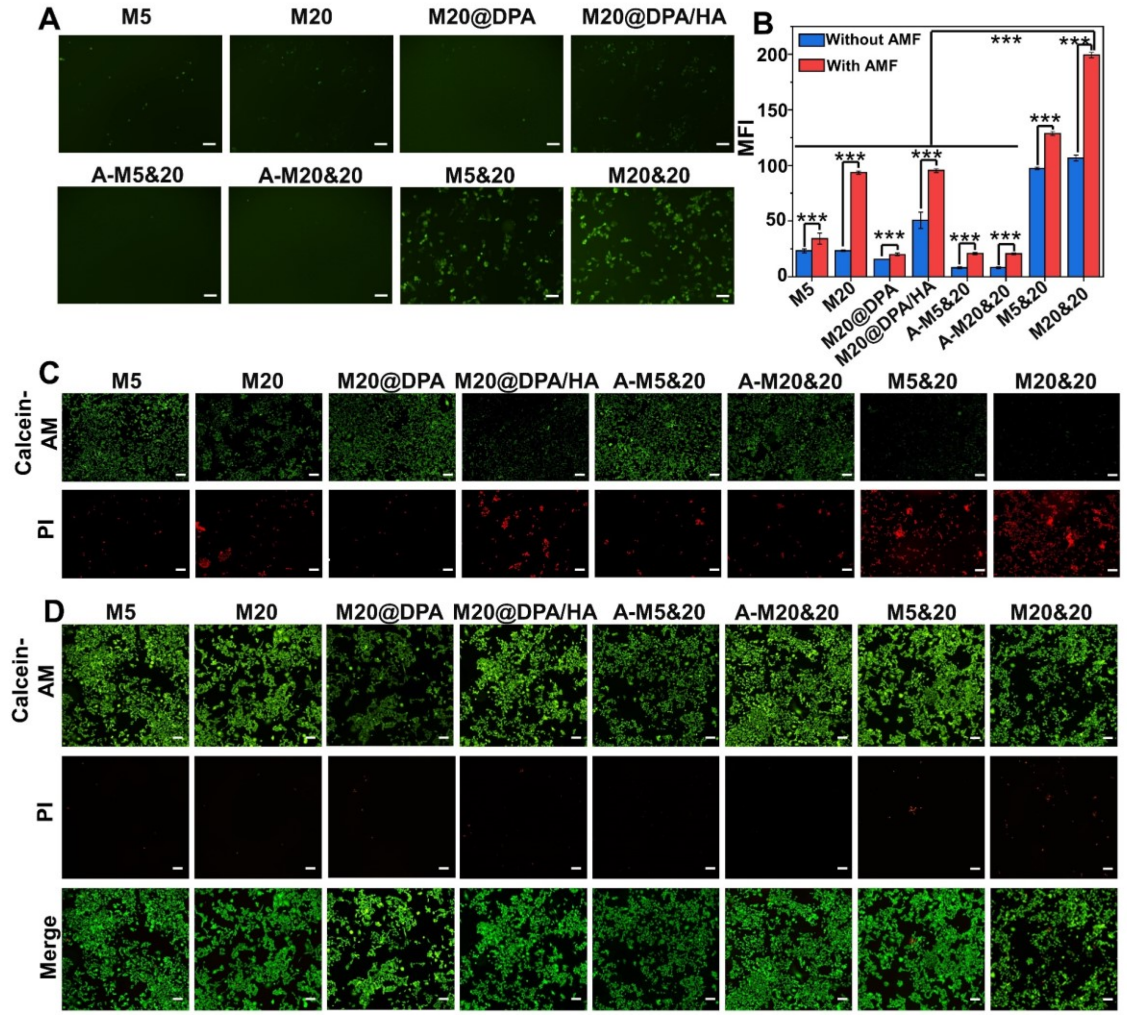

Figure S7. Killing effect of tumor cells on 4T1 by different MNPs. (A) Generation of ROS after 24 h co-incubation with different MNPs without AMF by fluorescent probe (DCFH-DA assay) staining. (B) Semi-quantitative analysis of ROS generation by MFI of fluorescent probe. (C) Live/dead staining with calcein-AM and PI after co-incubation with different MNPs under AMF. (D) Live/dead staining with calcein-AM and PI after co-incubation with different MNPs in the absence of AMF. Scale bars: 100 μm. *** $P < 0.001$.

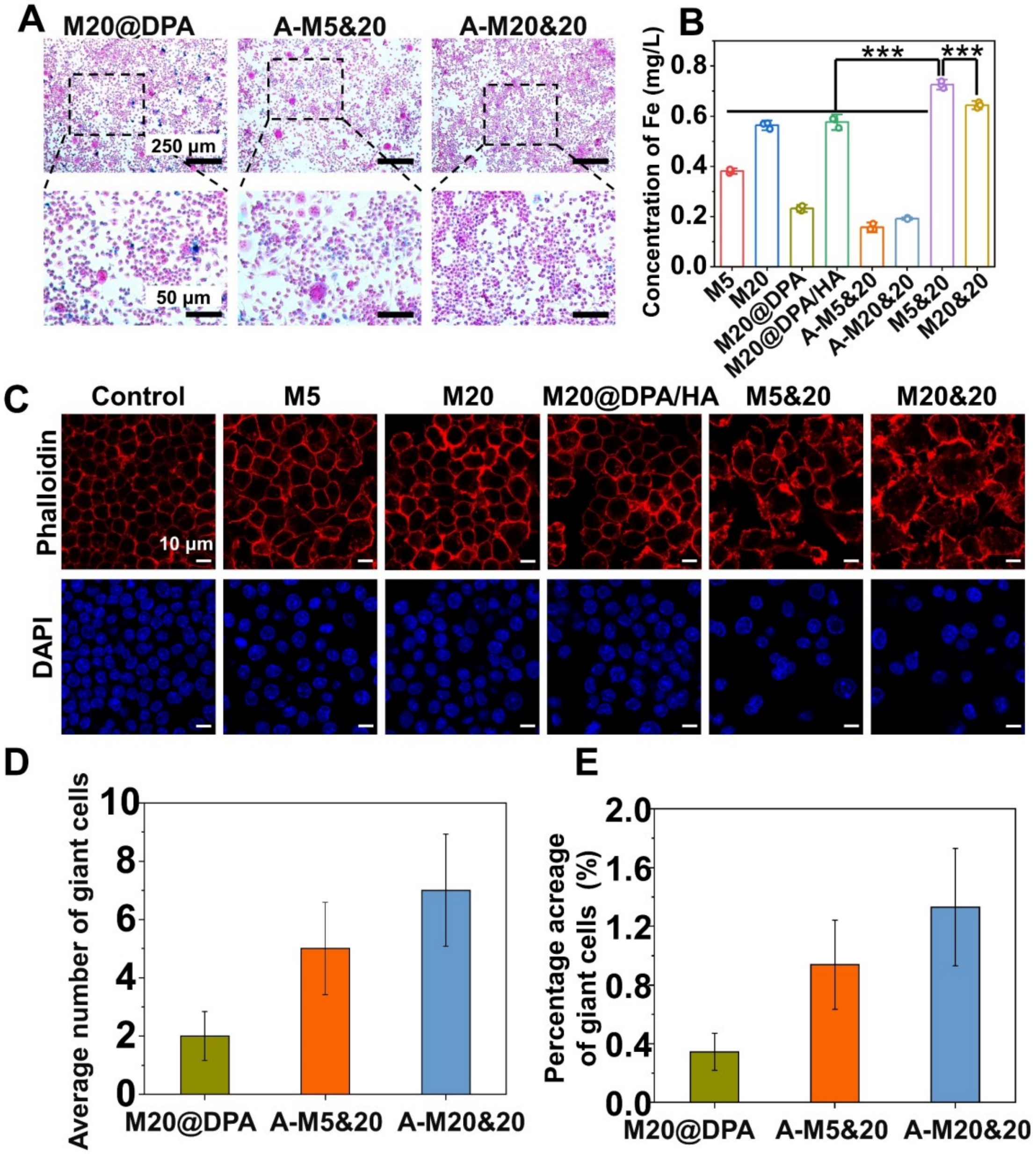

Figure S8. Immune activation in giant cells formation after treated with different MNPs. (A) Cellular uptake of different MNPs (M20@DPA, A-M5&20, A-M20&20) after 24 h incubation on RAW264.7 by Prussian blue staining. Scale bars: 250 and 50 μm, respectively. (B) Intracellular Fe concentrations of different MNPs, detected by ICP-OES analysis after 24 h incubation. (C) Detection of giant cells formation in RAW264.7 by

cytoskeleton fluorescence staining with phalloidin (red) and DAPI (blue) after different treatments. Scale bars: 10 μm. Average number (D) and percentage acreage (E) of the giant cells, calculated from the Prussian blue staining in Figure S8 A. *** $P < 0.001$.

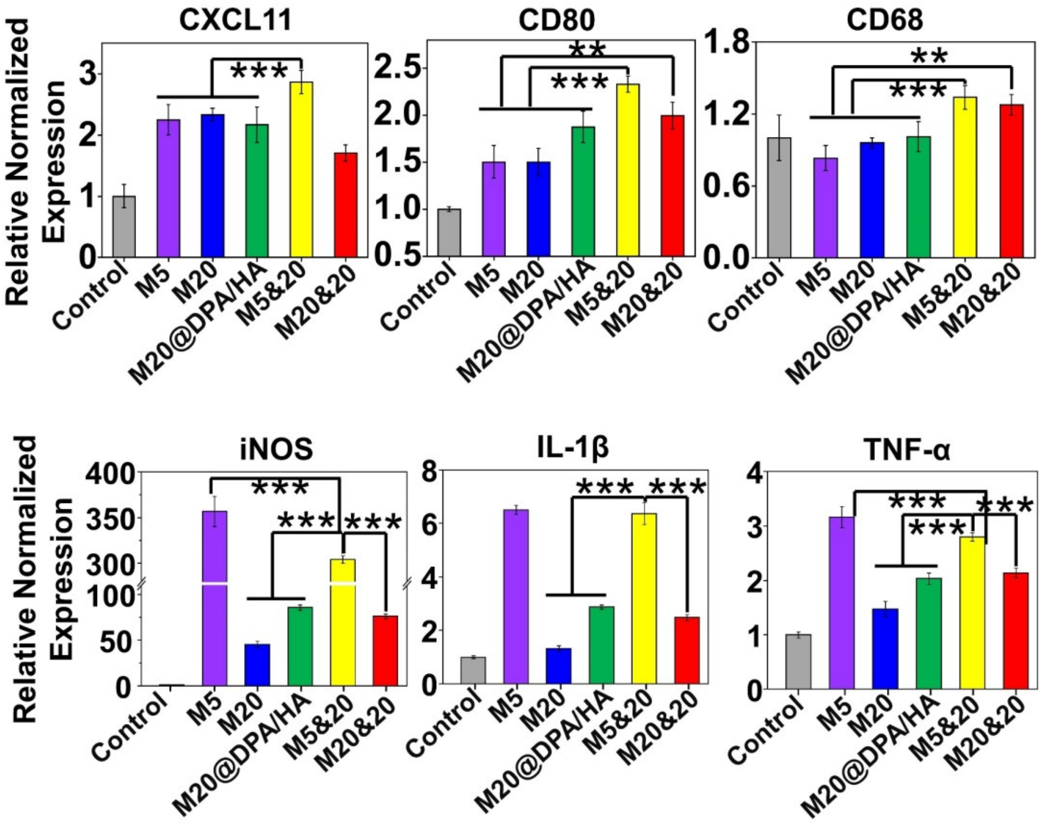

Figure S9. M1 polarization-related gene expression on RAW264.7 cells after treated with different MNPs for 24 h. ** $P < 0.01$, *** $P < 0.001$.

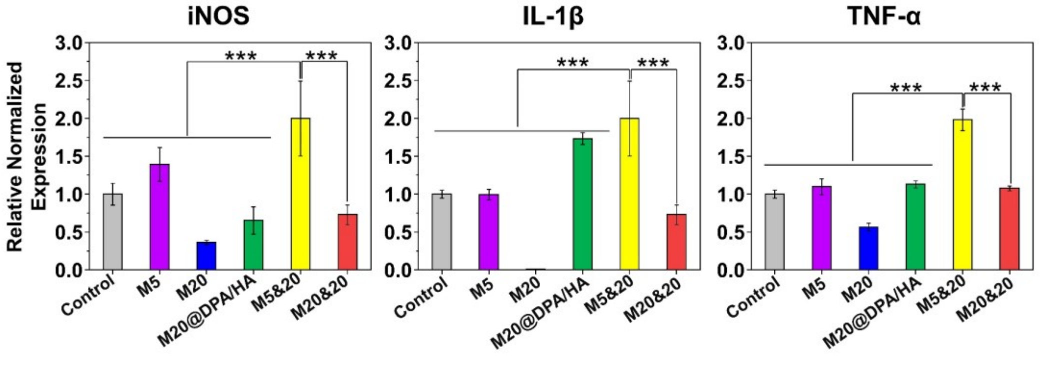

Figure S10. M1 polarization-related gene expression on RAW264.7 cells treated with AMF for 20 min after treated by different MNPs for 24 h. *** P < 0.001.

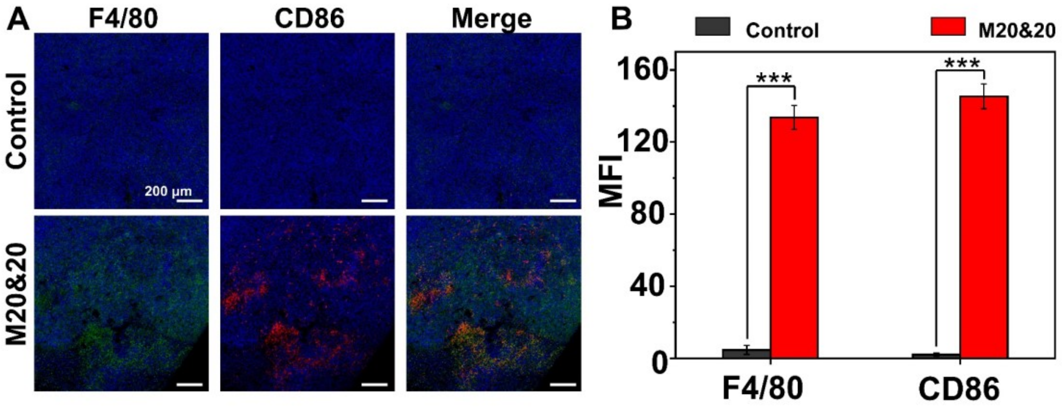

Figure S11. (A) Immunofluorescence (IF) staining images of F4/80 and CD86 on tumor tissue sections in control and M20&20 treated group, respectively. (B) MFI of F4/80 and CD86 calculated from the left CLSM images. *** P < 0.001.

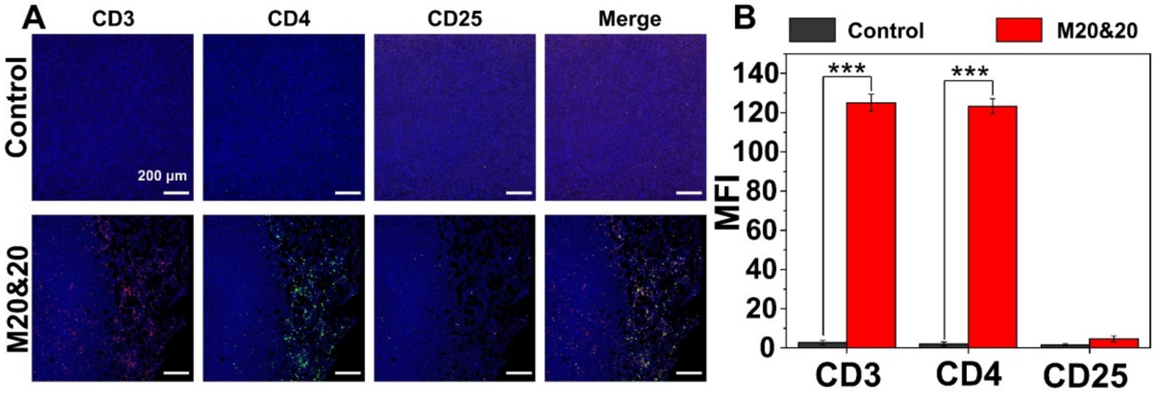

Figure S12. (A) Immunofluorescence (IF) staining images of CD3, CD4 and CD25 on tumor tissue sections in control and M20&20 treated group, respectively. (B) MFI of CD3, CD4 and CD25 calculated from the left IF images. *** P < 0.001.

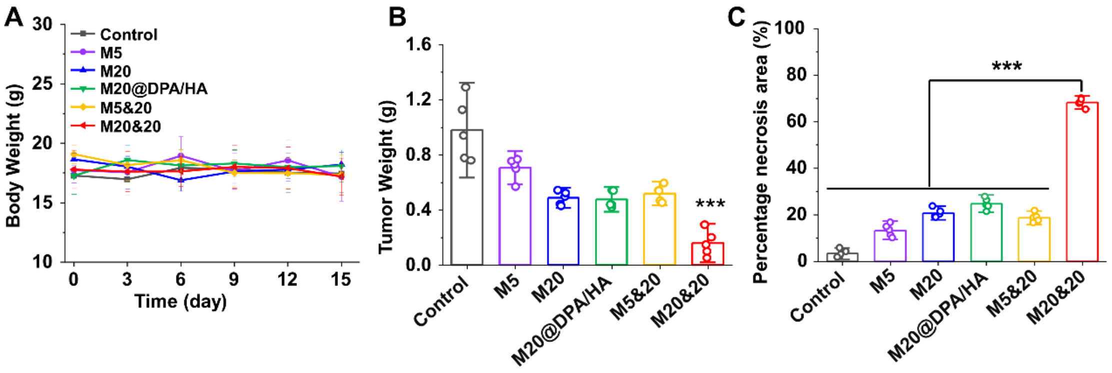

Figure S13. Therapeutic effects of intracellular aggregation of MNPs. (A) Body weight curves of mice treated with different MNPs during the treatment. (B) Tumor weight excised from mice post 15-day treatment. (C) The percentage of necrosis area (%) calculated from each tumor (n = 5). *** P < 0.001.

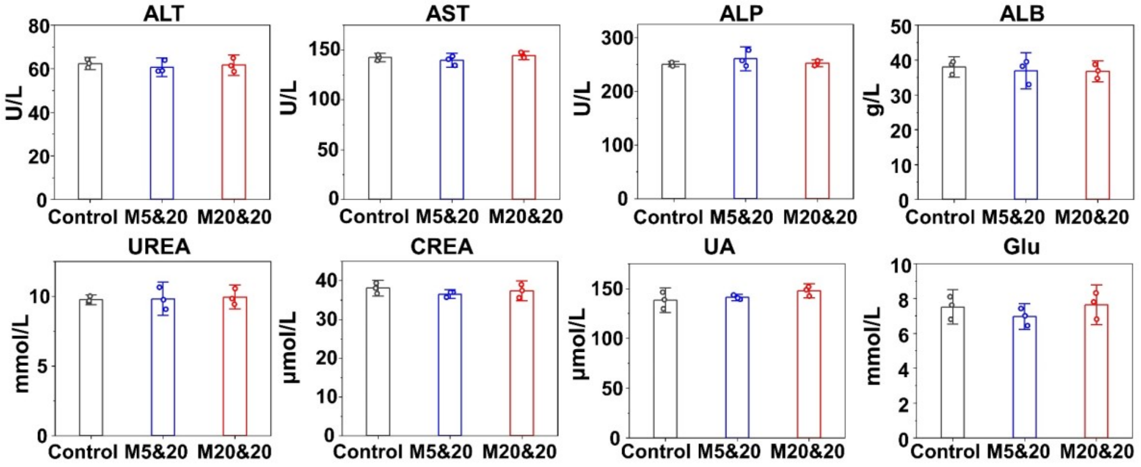

Figure S14. The blood biochemical indicators related to liver and kidney function after indicated treatment.

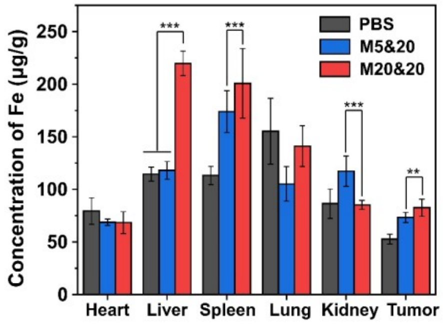

Figure S15. Biodistribution of M5&20 and M20&20 in 4T1 mouse mammary tumor model via 24 h-intravenous injection. *** P < 0.001.

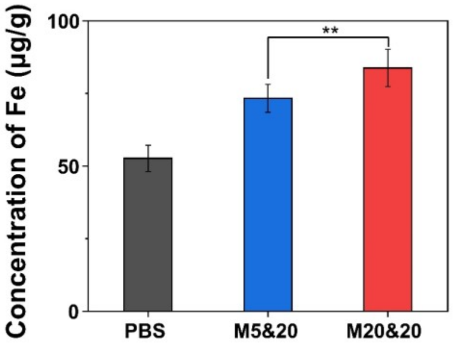

Figure S16. Fe concentration of tumor treated by M5&20 and M20&20 for 24 h. ** P < 0.01.

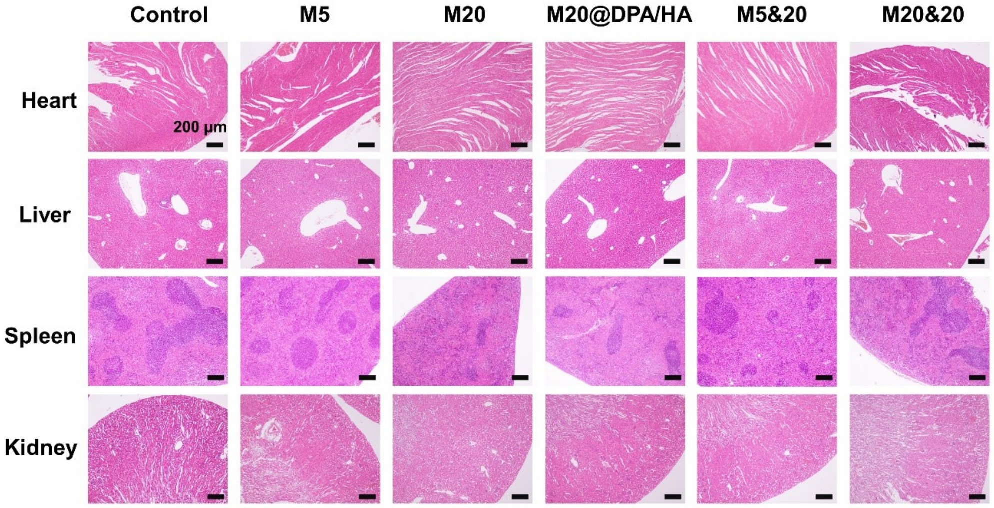

Figure S17. H&E analysis on sections of main organs at day 15 post treatment. Scale bars: 200 μm. Data are presented as mean ± SD (n = 5).

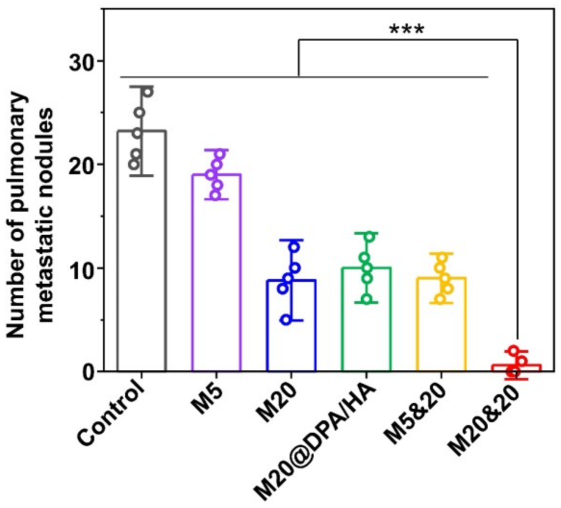

Figure S18. Number of lung metastatic nodules on day 15 after treated with different groups. *** P < 0.001.

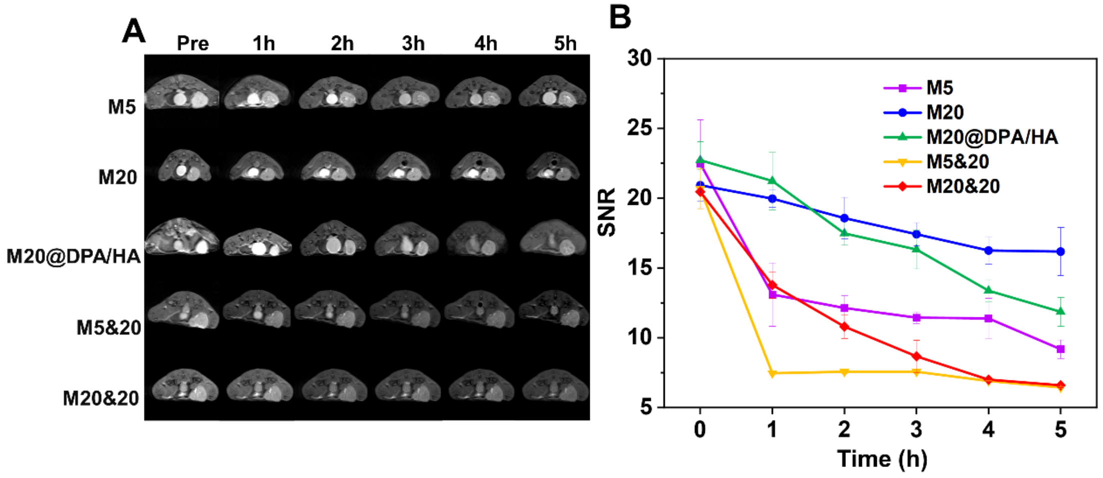

Figure S19. (A) In vivo T_2-weighted images of various MNPs at the predetermined time. (B) SNR values of various MNPs based on MRI signal over time.
